# Structure-Function Analysis of Barley NLR Immune Receptor MLA10 Reveals Its Cell Compartment Specific Activity in Cell Death and Disease Resistance

**DOI:** 10.1371/journal.ppat.1002752

**Published:** 2012-06-07

**Authors:** Shiwei Bai, Jie Liu, Cheng Chang, Ling Zhang, Takaki Maekawa, Qiuyun Wang, Wenkai Xiao, Yule Liu, Jijie Chai, Frank L. W. Takken, Paul Schulze-Lefert, Qian-Hua Shen

**Affiliations:** 1 State Key Laboratory of Plant Cell and Chromosome Engineering, Institute of Genetics and Developmental Biology, Chinese Academy of Sciences, Beijing, China; 2 Graduate University of Chinese Academy of Sciences, Beijing, China; 3 Department of Plant Microbe Interactions, Max-Planck Institut Pflanzenzüchtungsforschung, Cologne, Germany; 4 School of Life Sciences, Tsinghua University, Beijing, China; 5 Molecular Plant Pathology, Swammerdam Institute for Life Sciences, University of Amsterdam, Amsterdam, The Netherlands; 6 Centre for BioSystem Genomics, Wageningen, The Netherlands; The University of North Carolina at Chapel Hill, United States of America

## Abstract

Plant intracellular immune receptors comprise a large number of multi-domain proteins resembling animal NOD-like receptors (NLRs). Plant NLRs typically recognize isolate-specific pathogen-derived effectors, encoded by avirulence (*AVR*) genes, and trigger defense responses often associated with localized host cell death. The barley *MLA* gene is polymorphic in nature and encodes NLRs of the coiled-coil (CC)-NB-LRR type that each detects a cognate isolate-specific effector of the barley powdery mildew fungus. We report the systematic analyses of MLA10 activity in disease resistance and cell death signaling in barley and *Nicotiana benthamiana*. MLA10 CC domain-triggered cell death is regulated by highly conserved motifs in the CC and the NB-ARC domains and by the C-terminal LRR of the receptor. Enforced MLA10 subcellular localization, by tagging with a nuclear localization sequence (NLS) or a nuclear export sequence (NES), shows that MLA10 activity in cell death signaling is suppressed in the nucleus but enhanced in the cytoplasm. By contrast, nuclear localized MLA10 is sufficient to mediate disease resistance against powdery mildew fungus. MLA10 retention in the cytoplasm was achieved through attachment of a glucocorticoid receptor hormone-binding domain (GR), by which we reinforced the role of cytoplasmic MLA10 in cell death signaling. Together with our data showing an essential and sufficient nuclear MLA10 activity in disease resistance, this suggests a bifurcation of MLA10-triggered cell death and disease resistance signaling in a compartment-dependent manner.

## Author Summary

Plants utilize a multilayered immune system to protect themselves against pathogens. One layer of innate immunity is controlled by intracellular immune receptors called disease resistance (R) proteins. Plant R proteins are powerful molecules capable of triggering host cell suicide thereby restricting pathogen growth. Therefore, it is crucial for plants to control R protein activity in signaling cell death to avoid harmful autoimmune responses. The Barley *MLA* locus encodes a number of immune receptors that each recognizes a specific powdery mildew fungal strain. Upon pathogen recognition MLAs trigger host defenses concomitant with a rapid cell death response. We here show that MLA10 cell death-inducing activity is tightly regulated by conserved motifs located in two of its domains and by specific cellular chaperone components. Furthermore, we show distinct functions for the nuclear and cytoplasmic MLA10 pools in disease resistance and cell death signaling and provide evidence for a model uncoupling MLA10 cell death signaling from its disease resistance activity. Our results suggest that plant immune receptors integrate signals from multiple sub-cellular compartments to coordinate effective immune responses against pathogen attack.

## Introduction

Plants defend themselves against pathogens by mounting effective, spatiotemporally fine-tuned immune responses. Two major types of immune receptors are responsible for pathogen recognition and subsequent defense induction [1]. One class comprises membrane-localized pattern recognition receptors that launch PAMP/MAMP-triggered immunity (PTI/MTI) upon detection of pathogen/microbe-associated molecular pattern (PAMP/MAMP). The second type are intracellular disease resistance (R) proteins that trigger effector-triggered immunity (ETI) after recognition of pathogen delivered effector proteins [2], [3]. Although PTI/MTI and ETI share some signaling pathways and induce similar defense responses, ETI is more frequently associated with the hypersensitive response (HR). The HR is defined as a localized and rapid cell death response around attempted pathogen infection sites [4]–[7].

Plant intracellular R proteins structurally resembling mammalian NOD-like receptors (NLRs) are classified as STAND (signal transduction ATPases with numerous domains) NTPases [8]. This class of R proteins share a central conserved NB-ARC domain that is highly conserved in the human apoptotic regulator APAF-1, plant R proteins and CED-4 from *C. elegans* [9]. The NB-ARC domain is believed to act as a molecular switch that regulates STAND activity through binding and hydrolyzing nucleotides. Plant NB-ARC domains consist of three subdomains, called the NB, ARC1 and ARC2. All three subdomains contain many highly conserved motifs whose functions have been intensively studied [10], [11]. The P-loop (Walker A) motif in the NB for example, was shown to be required for nucleotide binding [12]–[14]. Mutations in the P-loop motif result in loss-of-function of several NB-LRR proteins [14]–[20]. The MHD motif in the ARC2 is predicted to act as a phosphate sensor and to be involved in nucleotide-dependent conformational changes [21]. Mutations in the MHD motif lead to autoactivation of many NB-LRR proteins [18], [21]–[25].

R proteins containing an NB-ARC domain are often referred to as NB-LRR proteins because most of them carry a C-terminal Leucine Rich Repeat domain (LRR). For many R proteins the LRR has been shown to determine pathogen recognition specificity [26]–[31]. Plant NB-LRR proteins are broadly subdivided into two subclasses: the TNL and the CNL type. This classification is based on their N-terminal domains, which either resembles a TOLL/interleukin-1 receptor (TIR) or forms a coiled-coil (CC) domain. The N-terminal TIR or CC domains are thought to mediate downstream signaling, eventually leading to induction of defense responses. Several lines of evidence are in support for such a signaling function for the N-terminal domain of R proteins [25], [31]–[35]. The CC domain is defined as a loosely conserved structure with at least three variants: CC, CC_EDVID_ or CC_R_. The CC_EDVID_ variant is named after the highly conserved “EDVID” motif, which is absent in the other two variants. The CC_R_ class is named after their founding members the *Arabidopsis* RPW8 proteins [36]–[38]. It is noteworthy that for Rx, a typical CC_EDVID_-NB-LRR subtype R protein, its central NB and not its N-terminal CC_EDVID_ domain is sufficient to induce cell death [39]. For the *Nicotiana benthamiana* NRG1 and the *Arabidopsis* ADR1 proteins, both belonging to the CC_R_-NB-LRR subtype, their CC_R_ domains alone can trigger cell death [38]. For RPS2, RPS5 and RPM1, all CC-NB-LRR proteins, it has been shown that their CC domains are required for ectopic cell death, but it is unknown whether their CCs alone are sufficient to induce defense signaling [16], [20], [24]. For barley MLA10 its CC_EDVID_ domain alone has been shown to be required and sufficient to induce cell death [25]. Taken together these data do not allow generalities or predictions on a signaling function for a particular CC domain or a CC domain type.

The subcellular localization of plant R proteins is important for their function. Several R proteins were shown to have a dynamic nucleo-cytoplasmic distribution and to accumulate in the nucleus in response to pathogen infection [40]–[42]. Although there are no discernable nuclear localization signals (NLS) in the barley MLA10 or the tobacco N proteins, their nuclear localization is essential for effective resistance [43], [44]. In addition, the activity of the *Arabidopsis* RPS4, RRS1-R and snc1 have also been associated with their nuclear localization [45]–[47]. Two recent studies on RPS4 revealed that the RPS4-EDS1 signaling complex exists in both nucleus and cytoplasm and each of these complexes can be activated by AvrRps4 [48], [49]. Strikingly, nuclear activation of RPS4 by enforced AvrRps4 nuclear localization uncouples the immune response from cell death signaling, however, full immunity requires nucleo-cytoplasmic coordination of both subcellular defense branches [49]. Studies on the potato Rx protein revealed that its nucleocytoplasmic distribution is balanced by its N-terminal and C-terminal domains and is facilitated by its interacting partner RanGAP2 [50], [51]. Intriguingly, hyperaccumulation of Rx in the nucleus blocked its cell death signaling and compromised resistance against PVX; whilst increasing the Rx cytoplasmic pool by overexpressing RanGAP2 resulted in potentiated defense signaling, leading to HR in the absence of PVX-CP and enhanced resistance to PVX [51].

The barley *MLA* locus is highly polymorphic in nature and has been subject to extensive functional diversification [52]. MLA encodes mainly allelic CNL-type R proteins, designated MLA1, MLA2 etc. Each MLA allele confers isolate-specific disease resistance against the barley powdery mildew fungus (*Blumeria graminis* f. sp. *Hordei*, *Bgh*). We have previously shown that MLA1 and MLA10 localize in the nucleus and cytoplasm before and after *Bgh* inoculation during compatible and incompatible interactions. Furthermore, in the nucleus MLA10 interacts with WRKY transcription factors that act as repressors of MAMP-triggered basal defenses; and importantly, the MLA10 nuclear pool is required for disease resistance against the *Bgh* [44]. MLA proteins harbor a largely invariant N-terminal CC domain of the CC_EDVID_ subtype. A recent study revealed that the CC of MLA forms a rod-shaped homodimer in solution and that a MLA dimer defines the minimal functional unit required for triggering cell death in barley and *N. benthamiana* [25].

In this study, we first conducted structure and function analyses of the MLA10 protein to better understand the regulation of the activity of the invariant CC domain in triggering cell death by using *Agrobacterium*-mediated transient expression in *N. benthamiana* leaves. We then show distinct functions for the nuclear and cytoplasmic MLA10 pools in disease resistance and cell death induction. Remarkably, manipulation of cytoplasmic or nuclear MLA10 levels reveals that the cytoplasmic pool of MLA10 alone is sufficient to induce cell-death, whereas nuclear-localized MLA10 is unable to induce cell-death, but is capable of conferring disease resistance.

## Results

### Only the CC Domain Has Cell Death Signaling Activity that Is Modulated by Other MLA10 Domains

**Figure 1:**
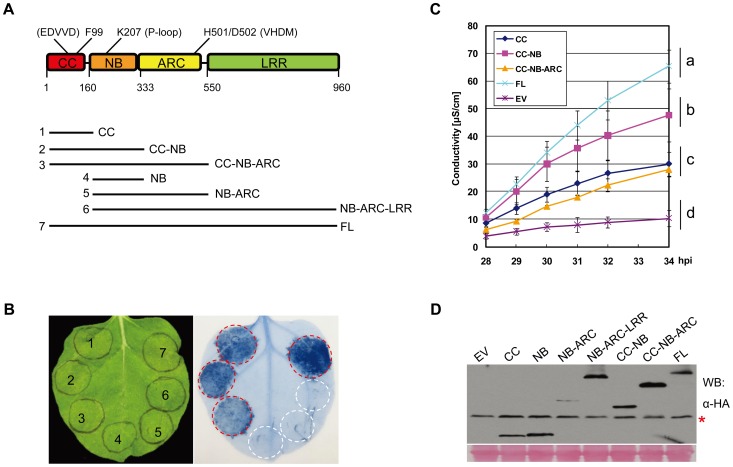
MLA10 CC is the cell death signaling domain whose activity is regulated by other domains. (**A**) Schematic diagram of the MLA10 domain structure and the derived fragments expressed in *N. benthamiana*. Individual domains of MLA10 are represented by colored boxes, and the relative positions of relevant amino acids and motifs (in parentheses) are indicated on top (upper panel). Lower panel: MLA10 fragments consisting only of the CC domain or the CC combined with other domains are drawn schematically in solid lines. All fragments were expressed as C-terminal -3×HA fusions in *N. benthamiana* leaves using *Agrobacterium*-mediated transient transformation (Agro-infiltration). (**B**) Analysis of cell death inducing activity of MLA10 fragments. MLA10 fragments or full-length(FL) protein fused with a C-terminal 3×HA tag were expressed by Agro-infiltration in *N. benthamiana* (left), and cell-death triggered by each protein was visualized by trypan blue staining at 42 hrs post Agro-infiltration (hpi) (right). Red circles indicate cell death; white circles indicate no obvious cell death in the infiltrated area. (**C**) Quantification of cell-death inducing activity of MLA10 fragments. Upon expression of the same fragment fusions as indicated in (**B**) by Agro-infiltration in *N. benthamiana*, electrolyte leakage was measured each hour from 28 to 34 hpi. Error bars were calculated from three replicates per time point and per construct. Experiments were done at least twice with similar results. Letters (a–d) represent groups with significant differences [*p*<0.05, Tukey's honest significant difference (HSD) test]. (**D**) Protein expression of MLA10 fragments. Total protein was extracted from *N. benthamiana* leaves at 40 hpi and MLA10-HA was detected by immunoblotting using an anti-HA antibody. Asterisk indicates non-specific signals throughout this article except specified. Ponceau staining of Rubisco small subunit was used to show equal loading throughout this article except specified. EV: empty vector.

The CC domain of MLA10 dimerizes in solution and defines the minimal functional unit required to trigger cell death [25]. To better understand the involvement of MLA10 domains in cell death induction a series of MLA10 fragments was generated. These fragments include the CC, NB, NB-ARC and NB-ARC-LRR alone, and the CC combined with one or multiple domains: CC-NB, CC-NB-ARC and CC-NB-ARC-LRR, all fused with 3×HA tag (Figure 1A). Upon expression of these fragments in *N. benthamiana* leaves and subsequent trypan blue staining or electrolyte leakage measurements, we observed a cell death phenotype by expression of the CC domain alone or CC domain-containing fragments (CC-NB, CC-NB-ARC and full-length [FL] MLA10). We never observed a cell death phenotype after expressing MLA10 fragments lacking the CC, i.e. NB, NB-ARC or NB-ARC-LRR (Figure 1B, 1C). All fusion proteins accumulated to detectable levels as revealed by immunoblotting (Figure 1D). To make sure that the C-terminal tags used here or in experiments below do not interfere with functions, we tested cell death activity of CC or CC-NB fused to different types of tag (Figure S1). Cell death activity of CC was not affected by small tags such as 3×HA or 3×Myc, whilst the function of CC-NB retained when fused to even larger tags, like YFP (Figure S1). These data indicate that only the CC domain can initiate the cell death response. When comparing the individual CC-containing fragments, we found that the CC-NB reproducibly triggered the strongest cell death response. The CC and CC-NB-ARC triggered comparatively weaker responses, suggesting that the NB positively regulates cell death whereas the ARC domain might exert a negatively regulatory effect (Figure 1B, 1C). Notably, MLA10 FL protein triggered an even stronger cell death response when compared to that of the CC-NB (Figure 1B, 1C), indicating that the FL protein is partially auto-active in the *N. benthamiana* heterologous expression system. In summary, the CC domain of MLA10 is necessary and sufficient for triggering cell death in *N. benthamiana*, and its activity is modulated by other MLA10 domains.

### Largely Invariant CC Sequence Is Critical for Its Cell Death-Inducing Activity

**Figure 2:**
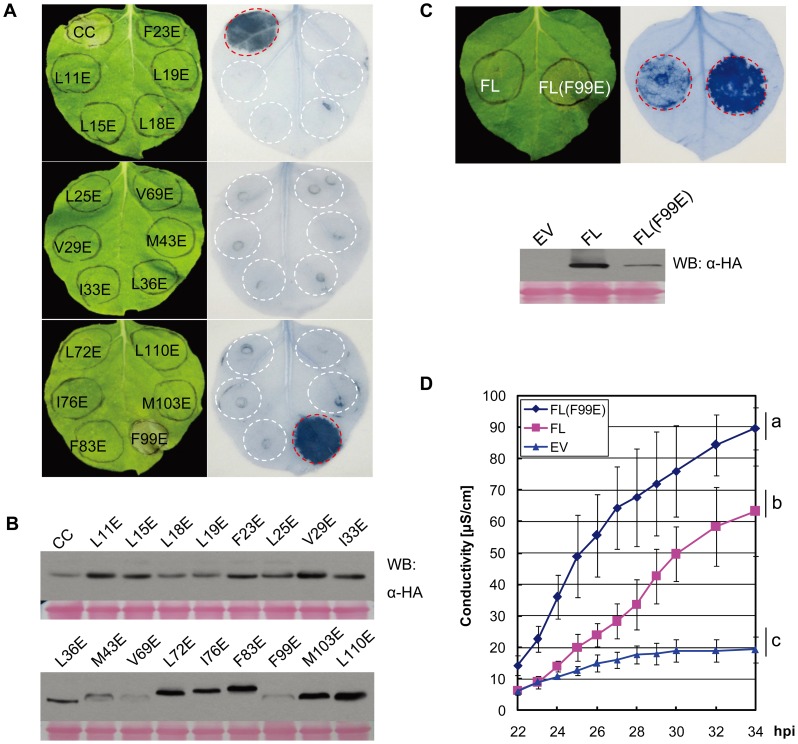
Glutamate substitution analyses in the MLA10 CC domain identify the F99E autoactive mutation. (**A**) Analysis of cell death inducing activity of MLA10 CC mutant variants. MLA10 CC wild-type or mutant variants harboring indicated amino acid substitution were expressed in *N. benthamiana* leaves, and cell death induced by each protein was visualized by trypan blue staining at 42 hpi (upper panel). (**B**) Protein expression levels of each CC variant shown by Western blotting. Proteins were extracted from *N. benthamiana* leaves at 40 hpi and detection was done by immunoblotting with anti-HA antibody. (**C**) Comparison of cell death inducing activity of MLA10 FL and the FL(F99E) variant. FL and FL(F99E) were expressed on the same *N. benthamiana* leaf, and the amount of cell death induced by each protein was shown by Trypan blue staining at 24 hpi (upper panel); protein expression levels of FL and FL(F99E) were shown by protein immunoblotting analysis using anti-HA antibody (bottom panel), protein extracts were obtained at ∼22 hpi. (**D**) Quantification of cell-death inducing activity of FL and FL(F99E). Upon expression of FL or FL(F99E) by Agro-infiltration in *N. benthamiana*, electrolyte leakage was measured each hour from 22 to 30 hpi, and then every two hours from 30 to 34 hpi; empty vector (EV) was included as a negative control. Error bars representing standard error (SE) were calculated from three replicates per time point and per construct. Similar experiments were repeated at least twice with similar results. Letters (a–c) represent significant differences [*p*<0.05, Tukey's honest significant difference (HSD) test].

**Figure 3:**
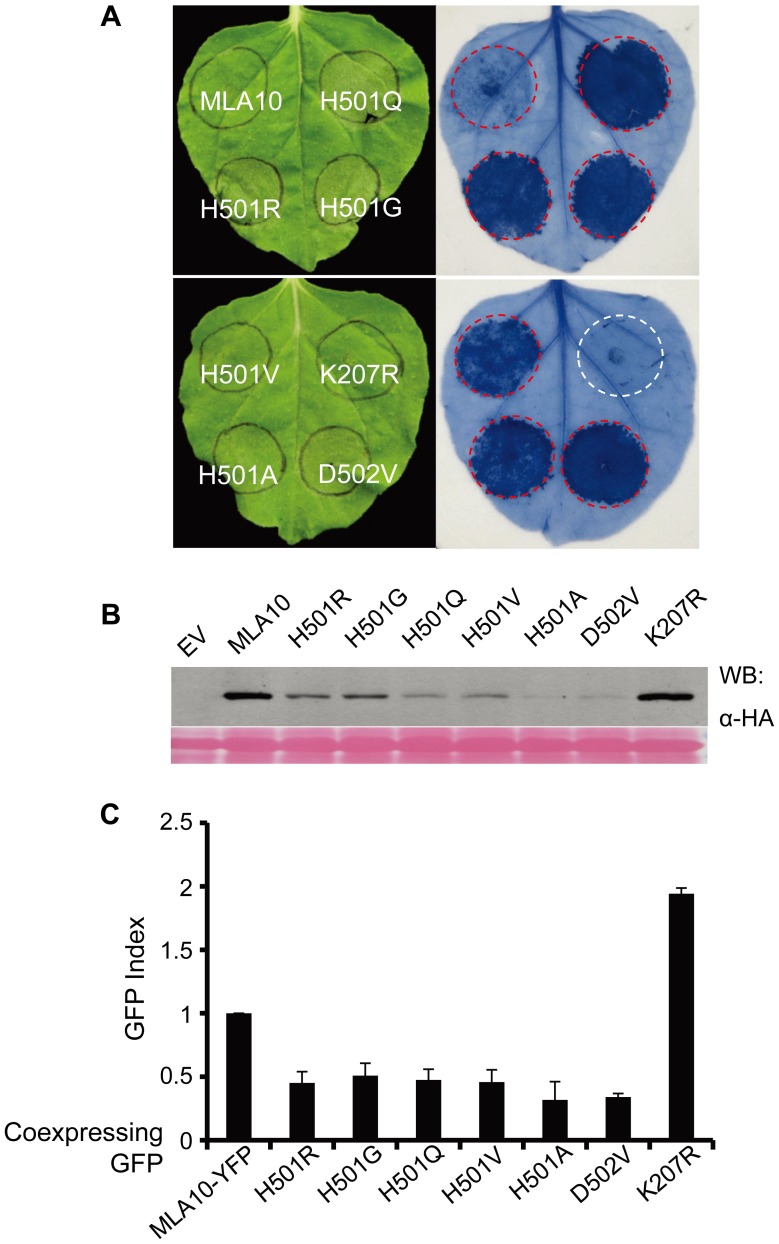
Cell death activity analyses of MLA10 MHD or P-loop mutant variants in *N. benthamiana* and barley. (**A**) Analysis of cell death Inducing activity of MLA10 WT and its mutant variants in *N. benthamiana*. Individual C-terminal 3×HA tagged MLA10 WT and mutant proteins were expressed by Agro-infiltration in *N. benthamiana*, and cell-death triggered by each protein was scored by trypan blue staining at 24 hpi. (**B**) Protein expression of MLA10 WT and its mutant variants. Total proteins were extracted from *N. benthamiana* leaves at 22 hpi and followed by immunoblotting. MLA10 was detected using an anti-HA antibody. (**C**) Analysis of cell death Inducing activity of MLA10 mutant variants in barley. Plasmids of MLA10 MHD motif mutants (H501R/G/Q/V/A, D502V) and a P-loop mutant (K207R) were co-expressed with a GFP maker plasmid in barley epidermal cells using biolistic delivery. The histogram bars represent the number of GFP expressing cell of each co-expression experiment standardized to the MLA10-YFP as control (see Materials and Methods). GFP expressing cells were scored as vital cells at 36–42 hrs post bombardment. The error bars represented SE of three representing experiments from more than five replicates.

Previously, seventeen amino acid residues lining the interior between the protomers of the MLA10 CC dimer were chosen for Glutamate substitutions to assess their contributions to dimer stability [25]. Substitutions of most of these 17 residues were shown to reduce MLA10 self-interaction. In addition, all of these substitutions, except L18E and F99E, compromised MLA10-mediated disease resistance [25]. Here we tested whether the same 17 substitutions affect the cell death-inducing activity of the MLA10 CC domain. We created CC variants each containing a single substitution, i.e. L11E, L15E, L18E, L19E, F23E, L25E, V29E, I33E, L36E, M43E, V69E, L72E, I76E, F83E, F99E, M103E, and L110E. These CC variants, fused to a 3×HA tag, were transiently expressed in *N. benthamiana*. Significantly, except for F99E, all substitutions diminished the cell death-inducing activity of the CC domain (Figure 2A). Most variants accumulated to similar levels (Figure 2B) although some showed reduced levels (M43E, V69 and F99E). Three variants showed a reduced mobility in the polyacrylamide gel (L72E, I76E and F83E) for unknown reasons (Figure 2B, lower panel). The F99E substitution is interesting because it is the only mutation that retained CC cell death signaling activity. It is also noteworthy that the L18E substitution abrogated the CC activity leading to cell death, whilst full-length MLA10 containing the same mutation was shown to retain wild-type-like disease resistance activity against *Bgh* [25]. To exclude that the loss of cell death activity of CC(L18E) might be due to altered subcellular protein localization, we constructed C-terminal YFP fusions of CC(L18E) and FL(L18E), whose expression were driven by 35S or Ubiquitin promoter for transient expression in *N. benthamiana* or barley leaf cells, respectively (Figure S2). As controls we generated CC(F83E)-YFP and FL(K207R)-YFP fusions, the F83E and the P-loop K207R mutation rendering MLA10 inactive (Figure 2A, [25], and below Figure 3). Upon transient expression and confocal imaging we detected for CC(L18E)-YFP or FL(L18E)-YFP in *N. benthamiana* or barley cells similar nucleocytoplasmic distribution patterns compared to the respective controls (Figure S2). Our data are consistent with previously proposed sequence constraints acting on the CC domain for MLA disease resistance [25], [52] and assign a critical role of the invariant CC sequence to its cell death-inducing activity. Because the tested substitutions are also needed for efficient CC dimer formation [25], our new data substantiate a previous suggestion that only the CC homodimer, which has a characteristic surface charge segregation, is capable to initiate the cell death response.

### The F99E Substitution in the CC Domain Autoactivates MLA10

To further characterize the F99E substitution, we introduced this alteration in the MLA10 FL context and compared the cell death-inducing activity of FL(F99E) to that of MLA10 FL in *N. benthamiana*. At 24 hrs post *Agrobacterium* delivery MLA10 FL triggered either no cell death or occasionally a weak cell death response, whereas FL(F99E) induced extensive cell death (Figure 2C), suggesting that the F99E mutation renders MLA10 highly autoactive. The lower protein accumulation level of FL(F99E) in the leaf tissue might indirectly reflect its elevated potency in triggering cell death or could be the consequence of a higher turnover compared to MLA10 FL (Figure 2C). Ion leakage quantification further confirmed the significantly elevated cell death-inducing activity of FL(99E) as compared to FL MLA10 (Figure 2D). In summary, these results identify F99E as a novel autoactive mutation located in the CC domain of MLA10.

### The Role of the MLA10 P-loop and MHD Motif in Triggering Cell Death

The P-loop and the MHD motif (VHDM in MLA) are two highly conserved motifs with important functions in regulating NB-LRR protein activity. To better understand the MLA cell death triggering activity, we generated MLA10 FL variants carrying point mutations in these two motifs. These include a P-loop variant FL(K207R) and six MHD variants, FL(H501R), FL(H501G), FL(H501Q), FL(H501V), FL(H501A) and FL(D502V). The MLA10 variants were each fused to a C-terminal 3×HA tag and their expression was driven by the 35S promoter. After *Agrobacterium* infiltration in *N. benthamiana* leaves, the cell-death phenotype for the variants was scored at 24 hrs post infiltration (hpi) by trypan blue staining. At this time point MLA10 FL normally does not trigger cell death although occasionally some cell death could be observed (Figure 3A, and below). By contrast, all MHD variants induced massive cell death, whereas the K207R P-loop mutant did not trigger any cell death at this or at later time points. These results indicate that the MHD mutations confer autoactivity of the receptor whereas the P-loop mutation abolishes its cell death activity. Immunoblotting showed that all fusion proteins accumulated to detectable levels in leaf tissue (Fig. 3B). The lower protein accumulation level observed for the MHD variants, compared to WT MLA10 and the K207R variant, likely reflects their stronger cell death-inducing activity or a higher turnover.

Since *N. benthamiana* is a heterologous expression system for barley MLA10, we wondered whether the cell death phenotype represents a genuine cell death signaling activity of the protein variants. Therefore, we tested the activity of the same set of MLA10 variants in barley using a transient gene expression assay based on DNA-coated gold particle delivery to single plant cells [28]. In this assay we quantified the number of barley leaf epidermal cells that express a green fluorescent protein (GFP) marker 36 to 40 hrs post co-delivery of a plasmid expressing the individual MLA10 variants under the control of the ubiquitin promoter. A higher or lower GFP index compared to the wild-type MLA10-YFP fusion protein (index defined as 1.0) indicates a weaker or stronger cell death-inducing activity for a given MLA variant, respectively (Figure 3C). Expression of the FL(K207R) P-loop variant resulted in a two-fold increase of GFP expressing cells as compared to the wild-type MLA10-YFP fusion [44] (Figure 3C), indicating that MLA10 cell death-inducing activity in barley requires the P-loop motif. In contrast, expression of all MHD variants led to a markedly reduction of GFP expressing cells by more than one half as compared to the MLA10-YFP fusion (Figure 3C), indicating that the MHD variants triggered autoactivation also in barley. The FL(H501A) and FL(D502V) are likely the two most active variants because their expression reproducibly resulted in fewer GFP expressing cells than the other variants, which is consistent with the lowest protein accumulation level for these two variants in *N. benthamiana* (Figure 3B, 3C).

In summary, we demonstrated that an MLA10 P-loop mutant lost its cell death-inducing activity whereas MLA10 MHD variants triggered massive effector-independent cell death, i.e. autoactivation, in both barley and *N. benthamiana*. The functional conservation of MLA10 cell death-inducing activity in both plant species suggests that the respective signaling components are likely evolutionarily conserved across monocotyledonous and dicotyledonous plants.

### MLA10 Cell Death-Inducing Activity Is Tightly Regulated

**Figure 4:**
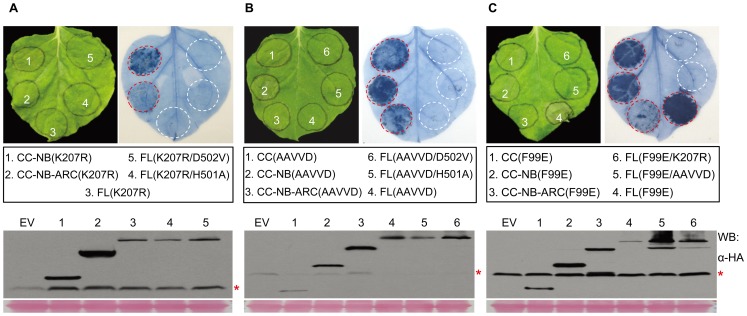
Tightly regulated MLA10 cell death inducing activity. (**A**) Analysis of cell death triggering activity of MLA10 variants harboring the K207R P-loop mutation. The indicated N-terminal MLA10 fragments containing the K207R mutation, or FL variants harboring K207R alone or K207R combined with an MHD mutation (H501, D502V), were expressed in *N. benthamiana* leaves by Agro-infiltration. Cell death triggered by each fusion was assessed by trypan blue staining at 42 hpi (upper panel). Protein expression levels of indicated MLA10 mutant fragments are shown (lower panel). Proteins were extracted at 40 hpi and detection was done by immunoblotting with anti-HA antibody. (**B**) Analysis of cell death activity of MLA10 variants harboring mutations in the EDVID motif. The EDVID motif (EDVVD in MLA10) was mutated to AAVVD, and the indicated MLA10 fragments or FL variants harboring indicated mutation(s) were expressed in *N. benthamiana* leaves by Agro-infiltration. Cell death triggered by each variant was assessed by trypan blue staining at 42 hpi (upper panel). Protein expression levels of indicated MLA10 mutant fragments are shown (lower panel). Proteins were extracted at 40 hpi and detection was done by immunoblotting with anti-HA antibody. (**C**) Analysis of cell death activity of F99E containing MLA10 fragments or FL variants. Indicated MLA10 fragments or FL variants harboring indicated mutation(s) were expressed in *N. benthamiana* leaves by Agro-infiltration. Cell death triggered by each protein was scored by trypan blue staining at 42 hpi (upper panel); Protein were extracts from *N. benthamiana* leaves and subjected to immunoblotting with anti-HA antibody (lower panel).

To further examine the regulation of the MLA10-mediated cell death activity by the P-loop motif, we introduced the K207R P-loop mutation into the MLA10 N-terminal fragments (CC-NB, CC-NB-ARC), and also combined this mutation with two autoactive MHD mutations (H501A or D502V) in MLA10 FL. The resulting K207R containing variants were then assessed for their cell death-inducing activity in *N. benthamiana* (Figure 4A). Unexpectedly, both CC-NB(K207R) and CC-NB-ARC(K207R) triggered cell death, and similar to the CC-NB and CC-NB-ARC fragments described above, the activity of the latter is weaker (Figure 4A, and 1B). However, just as the FL(K207R), the two FL variants, FL(K207R/H501A) and FL(K207R/D502V), were no longer able to induce cell death (Figure 4A). This indicates that the loss-of-function P-loop motif mutation in the receptor counteracts the autoactivating effect of the MHD motif mutations.

The MLA R proteins harbor an invariant N-terminal CC_EDVID_ domain containing the conserved “EDVID” motif (EDVVD in MLA10). Based on structure modeling it was proposed that the EDVID motif in MLA10 might be important for receptor post-activation signaling [25]. To gain more insight about its potential function, we mutated the EDVVD sequence to AAVVD in three MLA10 N-terminal fragments: CC, CC-NB and CC-NB-ARC, and in MLA10 FL, and also combined this mutation with two autoactive MHD mutations (H501A or D502V) in MLA10 FL, respectively (Figure 4B). Interestingly, cell death-inducing activity analyses showed that mutated N-terminal fragments, CC(AAVVD), CC-NB(AAVVD) and CC-NB-ARC(AAVVD), retained their cell death-inducing ability as their activity was comparable to that of the CC, CC-NB and CC-NB-ARC, respectively (Figure 4B, 1B). In contrast, the full-length variants, FL(AAVVD), FL(AAVVD/H501A) and FL(AAVVD/D502V), were unable to induce any cell death (Figure 4B). These results indicate that MLA10 FL protein, irrespective of WT or MHD variants, requires an intact EDVID motif for triggering cell death. Taken together, these results show that MLA10 N-terminal fragments (i.e. CC, CC-NB or CC-NB-ARC), lacking at least the C-terminal LRR, are capable of triggering EDVID and P-loop independent cell death. In contrast, MLA10 FL or FL MHD mutants fully depend on an intact EDVID and P-loop motif to trigger cell death.

To further test the requirement of EDVID and P-loop motif for another autoactive mutant, we utilized the newly identified F99E autoactive variant that carries a mutation in the N-terminal CC (Figure 2). The F99E substitution was combined in FL MLA with the K207R and AAVVD mutations, respectively, and the resulting FL(F99E/AAVVD) and FL(F99E/K207R) variants were compared for cell death activity to a series of controls, including CC(F99E), CC-NB(F99E), CC-NB-ARC(F99E) and FL(F99E) (Figure 4C, right panel). At 48 hpi, the F99E containing MLA10 N-terminal fragments, i.e. CC(F99E), CC-NB(F99E) triggered obvious cell death while CC-NB-ARC(F99E) triggered a weaker but still discernable cell death. These observations again indicate the negative modulatory effect the ARC domain has on the CC-mediated cell death activity. Notably, the F99E autoactivating effect was strongest in the context of the full-length receptor FL(F99E). Remarkably, FL(F99E/AAVVD) and FL(F99E/K207R) did not trigger any visible cell death (Figure 4C, right panel). These findings indicate that autoactivation in MLA10 FL F99E-triggered cell death requires an intact EDVID and P-loop motif.

It has been shown that allelic MLA proteins differentially engage components of the intracellular chaperone machinery (e.g. cytosolic HSP90, SGT1 and RAR1) to confer race-specific disease resistance against the barley powdery mildew fungus [28], [53]–[56]. For example, Barley Stripe Mosaic Virus (BSMV) mediated silencing of *RAR1*, *SGT1* and *HSP90* led to breakdown of MLA13-mediated resistance against *Bgh* [56]. The role of RAR1 in controlling MLA steady state levels has been demonstrated for both MLA1 and MLA6 [54]. To assess whether these chaperone components are also required for MLA10-mediated cell death, we used Tobacco Rattle Virus (TRV)-mediated VIGS to knock down the expression of the homologous chaperone components in *N. benthamiana* [57], [58]. Subsequently, the MLA10 cell death inducing activity was evaluated in these silenced lines using *Agrobacterium*-mediated expression (Figure S3). We found that *NbSGT1* silencing eliminated the cell death-triggering activity of MLA10 N-terminal fragments, including MLA10 CC, CC-NB and CC-NB-ARC, as well as the FL protein and the two MHD mutant variants tested (H501A and D502V), suggesting an essential role of *Nb*SGT1 in MLA10-triggered cell death in this heterologous expression system (Figure S3, 2^nd^ lane). In contrast to *NbSGT1*, *Nb*RAR1 silencing did not affect the cell death-inducing activity for any of these MLA fragments or the FL proteins, indicating that RAR1 is not essential for the MLA10-mediated cell death response (Figure S3, 3^rd^ lane). Interestingly, *Nb*HSP90 is likely required for the MLA10-mediated cell death, but this requirement could be partially overcome by the expression of MHD mutant variants that confer a strong receptor autoactivation (Figure S3, 4^th^ lane).

### Distinct Nuclear and Cytoplasmic MLA10 Activities in Disease Resistance and Cell Death Signaling

**Figure 5:**
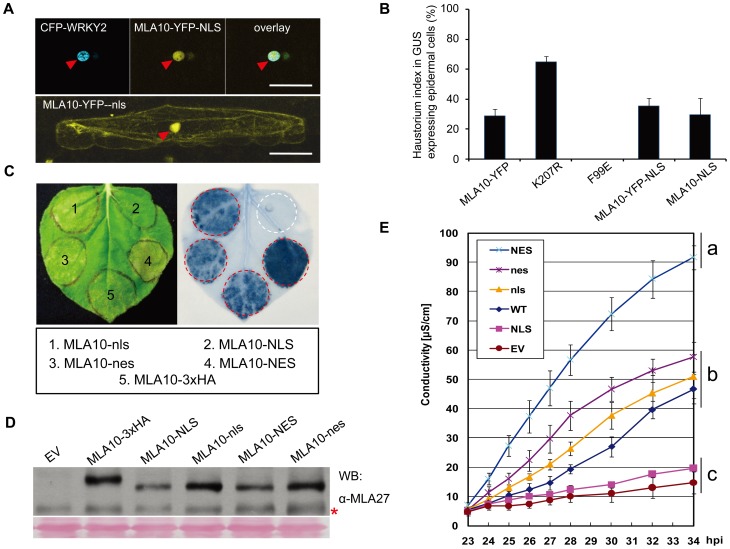
Manipulation of MLA10 subcellular localization and the relevance to MLA10 cell death signaling and disease resistance. (**A**) Confocal images of barley leaf epidermal cells expressing MLA10 fusion proteins. Indicated fusion proteins were expressed in barley leaf epidermal cells upon biolistic delivery, the confocal images were taken at 36 hrs post bombardment. Upper panel: A representative barley cell coexpressing MLA10-YFP-NLS and a nucleus marker CFP-WRKY2 (2D *z* plane). Lower panel: a cell expressing MLA10-YFP-nls alone (2D *z* plane). NLS: nuclear localization signal; nls: mutated nuclear localization signal. Arrowheads mark the nucleus and scale bar is 50 µm. (**B**) Analyses of disease resistance activity of MLA10 fusions or mutant variants. Relative single cell resistance/susceptibility is shown by fungal haustorium index upon biolistic delivery of plasmids expressing indicated protein and a GUS reporter into the barley leaf epidermal cells of a susceptible barley line (Golden promise). Bombarded leaves were inoculated with *B. graminis* fungal spores expressing AVR_A10_, and the fungal haustorium index was microscopically scored at 36 hrs post spore inoculation. Histogram bar represents average of three independent experiments and error bar represents SD. In the case of MLA10(F99E) expression, the number of cells expressing GUS reporter were extremely low. (**C**) Analysis of cell death triggering activity of MLA10 fusion proteins. Indicated MLA10 fusion proteins were expressed in *N. benthamiana* leaves by Agro-infiltration, and cell-death triggered by each fusion protein was scored by trypan blue staining at 40 hpi. NES: nuclear exclusion signal; nes: mutated nuclear exclusion signal. (**D**) Protein expression of indicated MLA10 fusions. Proteins were extracted at 23 hpi and MLA was detected by immunoblotting using an anti-MLA27 monoclonal antibody. Asterisk indicates non-specific signals. (**E**) Quantification of cell-death inducing activity of MLA10 fusion proteins. Upon expression of indicated MLA10 fusion proteins by Agro-infiltration in *N. benthamiana*, ion leakage was measured each hour from 23 to 34 hpi. Error bars (SE) were calculated from three replicates per time point and construct. Experiment was done at least twice with similar result. Letters (a–c) represent groups with significant differences [*p*<0.05, Tukey's honest significant difference (HSD) test].

We have previously shown that MLA10 locates to both the cytoplasm and the nucleus, and importantly, the nuclear pool is required for its disease resistance [44]. In this study, we are interested in how MLA10 subcellular partitioning relates to its cell death-inducing activity and disease resistance function. First we generated two fusion constructs, MLA10-YFP-NLS and MLA10-YFP-nls, whose expression is driven by the Ubiquitin promoter (NLS is a nuclear localization sequence from SV40 virus, while ‘nls’ is a mutated NLS and serves as a negative control; [59]). Upon expression of MLA10-YFP-NLS in barley leaf epidermal cells, YFP-derived fluorescence was exclusively detected in the nucleus. The YFP signal overlapped with that of a nuclear marker protein, CFP-WRKY2 [44], confirming its nuclear localization. The fluorescence signal of the MLA10-YFP-nls variant clearly partitioned to both nucleus and cytoplasm, similar to that of MLA10-YFP (Figure 5A, and [44]). This experiment demonstrated that the NLS functions to localize MLA10-YFP into plant cell nuclei. We wondered whether the nuclear localized MLA10-YFP-NLS confers disease resistance against *Bgh*. To address this, we utilized the single-cell transient expression assay [44], in which we delivered gold particles coated with DNA plasmids coexpressing a GUS reporter and either MLA10-YFP-NLS or MLA10-NLS into barley epidermal cells. Subsequently, the frequency of fungal structures formed inside the transformed cells (haustorium index; [44]) was scored upon inoculation with a *Bgh* race carrying the cognate AVR_A10_ effector. Included in these experiments are control plasmids expressing MLA10-YFP and the MLA10(K207R) P-loop mutant, respectively (Figure 5B). As expected the MLA10-YFP fusion conferred resistance against *Bgh* infection, resulting in an haustorium index around 29%, similar to our previous study [44]. The K207R P-loop mutation reduced MLA10 resistance activity as indicated by a haustorium index as high as 65%. In contrast, MLA10-YFP-NLS and MLA10-NLS fusions mediated similar levels of *Bgh* resistance as the MLA10-YFP control, with haustorium indexes of 35% and 30% respectively (Figure 5B). These data indicate that MLA10, forced to localize into the nucleus, is capable of triggering resistance to *Bgh*. Previously, we observed a haustorium index of 52% for the MLA10-YFP-NES fusion, which was undetectable in the nucleus. Since a similar haustorium index was obtained for the empty vector control, this indicates its loss-of-function against *Bgh* [44]. Together, these data suggest that the MLA10 nuclear pool is required for and capable of mediating disease resistance against the *Bgh* fungus in barley.

In order to link MLA10-mediated cell death activity to a specific subcellular receptor pool, we switched to the *N. benthamiana* transient expression system. We made two pairs of MLA10 fusions, MLA10-NES *vs.* MLA10-nes (NES is a nuclear export sequence, ‘nes’ is a mutated nonfunctional NES; [44]) and MLA10-NLS *vs.* MLA10-nls. Expression of all MLA constructs was driven by the 35S promoter (Figure 5C). Remarkably, MLA10-NLS was unable to trigger cell death whereas MLA10-nls retained cell death-inducing activity. Furthermore, expression of MLA10-NES triggered a cell death response that was more severe than the one initiated by MLA10-nes, MLA10-nls, and MLA10-3×HA at 36–40 hpi (Figure 5C). The stronger cell death activity of MLA10-NES is not due to higher protein accumulation as our anti-MLA27 antibody detected similar accumulation levels for these fusion proteins (Figure 5D) (anti-MLA27 is a monoclonal antibody raised against purified MLA27 that most likely cross-reacts with a conserved epitope between MLA10 and MLA27). Independent ion leakage measurements confirmed the results obtained from the trypan blue staining assay and showed that the stronger cell death-inducing activity of MLA10-NES was detectable at even earlier time points, compared to the WT receptor, starting from around 24 hpi (Figure 5E). To corroborate these data obtained from *N. benthamiana*, we decided to test in barley the cell death inducing activity of MLA10-NES and MLA10-NLS through the transient cell death assay used above (Figure 3C), included in this assay as controls were the autoactive D502V variant and the K207R non-functional P-loop variants (Figure S4). Significantly, in barley epidermal cells we observed for MLA10-NLS little cell-death inducing activity similar as the K207R, by contrast, for MLA10-NES potentiated cell-death inducing activity similar as the D502V (Figure S4).

It is intriguing that MLA10-NLS does not trigger cell-death, while the same construct confers full *Bgh* resistance. One explanation might be that the receptor needs to be activated for cell-death induction. Since MLA10 mediate race-specific resistance to *Bgh* isolates expressing the cognate effector AVR_A10_ [60] we speculated that co-expression of AVR_A10_ in the nucleus might activate MLA10. Since the NLS variant, in contrast to the non-tagged MLA is not autoactive, this became testable. Therefore, we made an AVR_A10_-YFP fusion and examined its subcellular localization and ability to induce cell-death in *N. benthamiana* leaf cells upon Agro-infiltration. Confocal imaging revealed accumulation and distribution of the AVR_A10_-YFP fusion over both the nucleus and the cytoplasm (Figure S5, left panel). Expression of AVR_A10_-YFP alone did not induce cell-death, but also coexpression of AVR_A10_-YFP with MLA10-NLS did not induce cell-death in *N. benthamiana* (Figure S5, right panel).

**Figure 6:**
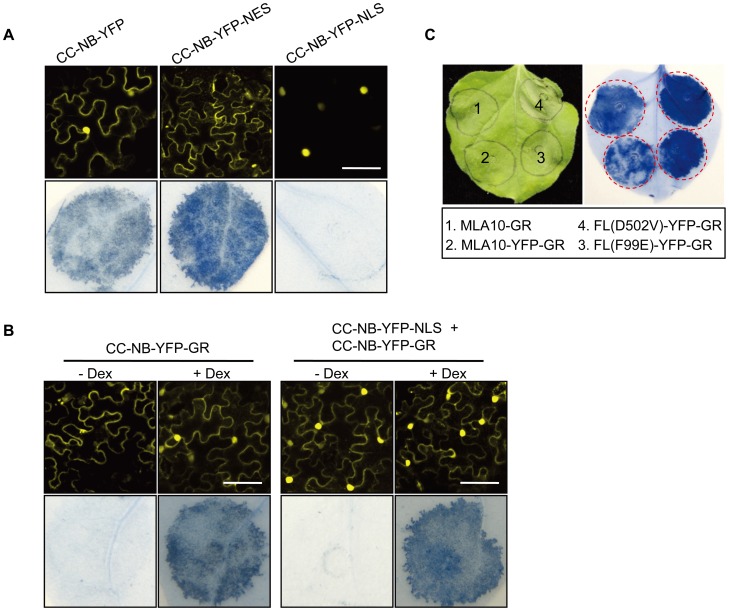
MLA10 CC-NB or full-length trigger cell death signaling in the cytoplasm. (**A**) Analysis of cell death inducing activity of MLA10 CC-NB fusion proteins. MLA10 CC-NB fusion proteins, CC-NB-YFP, CC-NB-YFP-NES and CC-NB-YFP-NLS, were expressed in *N. benthamiana* leaves by Agro-infiltration; confocal images were taken at ∼22 hpi (upper panel) and cell-death triggered by each fusion protein was scored by trypan blue staining at ∼48 hpi (lower panel). Scale bar is 50 µm. (**B**) Analysis of cell death phenotype upon expression of MLA10 CC-NB-YFP-GR or co-expression of MLA10 CC-NB-YFP-NLS and CC-NB-YFP-GR fusions before and after Dex treatment. The indicated fusion(s) were expressed in *N. benthamiana* leaves by Agro-infiltration. Buffer with/or without Dex was sprayed onto *N. benthamiana* leafs 20 hpi before the confocal images were taken (upper panel). The cell-death phenotype of each fusion protein was scored by trypan blue staining at ∼48 hrs post treatment with/or without Dex (lower panel). GR: Steroid binding domain of the mammalian glucocorticoid receptor. Scale bar is 50 µm. (**C**) Analysis of cell death activity of GR fusions of MLA10 WT and autoactive variants. Indicated MLA10-GR fusion, and the C-terminal YFP-GR fusions of MLA10 WT and two autoactive mutant variants were expressed in *N. benthamiana* leaves by Agro-infiltration. The cell-death phenotype was revealed by trypan blue staining at ∼24 hrs post infiltration without Dex induction.

To further study the subcellular localization of MLA10 in *N. benthamiana*, we constructed MLA10 CC-NB-YFP and MLA10-YFP fusions with a C-terminal YFP sequence. The expression of both constructs was driven by a 35S promoter. Upon Agro-infiltration in *N. benthamiana*, we compared the fluorescence signal as well as the cell death phenotype of CC-NB-YFP with MLA10-YFP. Confocal imaging revealed that both fusion proteins localized to the nucleus and the cytoplasm but the CC-NB variant had a brighter YFP fluorescence signal and a clearer nuclear localization than full-length MLA10, possibly due to differences in protein folding and/or protein turnover (Figure S6). Since both fusions triggered cell death, we decided to use CC-NB-YFP instead of MLA10-YFP in the following experiments. We fused the C-terminus of CC-NB-YFP to either an NES or NLS, and upon expression confocal imaging showed that in most cells the CC-NB-YFP-NES proteins localized to the cytoplasm, whilst the CC-NB-YFP-NLS proteins exclusively localized in the nuclei (Figure 6A, upper panel). Significantly, in cell death staining assays, CC-NB-YFP-NES induced a much stronger cell death phenotype as compared to the CC-NB-YFP fusion, whereas CC-NB-YFP-NLS did not trigger any cell death (Figure 6A, lower panel). All these fusion proteins accumulated to similar levels (Figure S7). These results are consistent with those obtained with the MLA10 full-length protein tagged with NES/NLS or nes/nls (compare to Figure 5C).

In summary, the nuclear MLA10 pool alone cannot induce cell death although it is sufficient to trigger disease resistance. Accelerated MLA10 exclusion from the nucleus and/or enhanced nucleocytoplasmic shuttling (i.e. in the NES tagged configuration) enhanced MLA10-mediated cell death activity, but was shown before to exhibit compromised MLA10-mediated disease resistance activity (Figure 1 in [44]). An unanswered question is whether the cytoplasmic MLA10 pool alone is sufficient to induce cell death.

### MLA10 FL Retained in Cytoplasm Does Trigger Cell Death Signaling

Because the mammalian glucocorticoid receptor (GR) with a hormone binding domain is folded into a larger HSP-containing complex in the cytosol, it is possible to use this domain to retain GR protein fusions in this cellular compartment and study their cytoplasmic functions [61]. This strategy has also been successfully used in plants [62]–[64]. Therefore, to particularly address whether the MLA10 cytoplasmic portion is sufficient for cell death signaling, we constructed CC-NB-YFP-GR fusions under the control of the 35S promoter for expression in *N. benthamiana*. Around 6 hrs after *Agrobacterium* infiltration we sprayed *N. benthamiana* leaves with a buffer with or without dexamethasone (Dex) and examined these leaves using confocal imaging after ∼14 hrs. In the controls without Dex treatment, the YFP fluorescence signals were largely restricted to the cytoplasm in the majority of cells; expectedly, after Dex treatment the YFP fluorescence signals were markedly intensified in most nuclei, indicating Dex-induced nuclear relocation of a portion of CC-NB-YFP-GR fusion (Figure 6B, left panel). In leaves expressing CC-NB-YFP-GR, without applying a Dex treatment, no obvious cell death could be observed, whereas upon Dex treatment cell death was markedly induced in CC-NB-YFP-GR expressing leaves. The extent of cell death for the latter was comparable to that of a CC-NB-YFP control (Figure 6A left, 6B left panel). Compared to the enhanced cell-death activity of CC-NB-YFP-NES, the lack of cell-death-inducing activity of CC-NB-YFP-GR in the absence of Dex induction could be explained by the need for a nuclear MLA fraction to exert this activity. To examine this possibility, we co-expressed the CC-NB-YFP-NLS and CC-NB-YFP-GR fusion proteins in the same cells to accomplish localization of the CC-NB-YFP in both the nucleus as well as in the cytoplasm (Figure 6B, right panel). Upon coexpression of CC-NB-YFP-NLS and CC-NB-YFP-GR, strong YFP signals could easily be detected in both the nucleus and the cytoplasm in the same cell in the absence of Dex, yet no obvious cell death was observed in the staining assays (Figure 6B, right panel). Again, upon Dex treatment, cell death was induced (Figure 6B, right panel). This finding indicates that, even in the presence of nuclear-localized CC-NB-YFP-NLS, cytoplasmically retained CC-NB-YFP-GR cannot induce cell death. Since the GR steroid-binding domain is folded by the large HSP-complex in the cytoplasm it is possible that the inability of the CC-NB-YFP-GR fusion to induce cell-death was due to steric hindrance by this chaperone complex. To investigate this possibility we made GR fusions to MLA10 FL and MLA10-YFP to have the endogenous 15 LRRs flanking the CC-NB to act as linker and thereby relieve potential hindrance. The F99E and D502V autoactive mutations were also introduced in MLA10-YFP-GR to make FL(F99E)-YFP-GR and FL(D502V)-YFP-GR fusion constructs. Upon Agro-infiltration and expression of the MLA10 FL fusions in *N. benthamiana* leaves MLA10-YFP-GR was retained in the cytoplasm in the absence of Dex induction, similar to CC-NB-YFP-GR (Figure S8, Figure 6B). Unexpectedly, in the absence of Dex MLA10-GR and MLA10-YFP-GR induced cell-death at similar levels, whereas the two autoactive fusions, FL(F99E)-YFP-GR and FL(D502V)-YFP-GR induced a stronger cell-death response as compared to the WT fusions (Figure 6C). These results suggest that MLA10 FL proteins retained in the cytoplasm are capable of triggering cell-death, while truncated forms lacking the LRR have to be released from the GR complex in order to signal cell death.

## Discussion

### The Role of the CC Domain and the Regulation of MLA10 Cell Death-Inducing Activity

Expression of MLA10 FL or CC containing fusions can induce AVR-independent cell death by *Agrobacterium*-mediated transformation in *N. benthamiana* leaf cells (Figure 1, 2, and 3). We believe that this partial auto-activation of MLA10 is unlikely due to an overexpression effect since we did not observe a direct correlation between protein stead-state levels and cell death activity (Figure 1 and 2C). Nevertheless, because for the FL receptor the cell death response was shown to be entirely dependent on an intact P-loop motif in the NB domain and the EDVID motif in the CC domain (Fig. 3 and 4), our data imply that cell death signaling *via* the CC domain is powered by receptor NTP hydrolysis and that pro-death signaling components are evolutionarily conserved between monocot and dicot plants.

The MLA proteins belong to the CC_EDVID_-NB-LRR subclass of CNLs, whose members share the conserved EDVID motif [37], [52]. Although the CC_EDVID_ of several CNLs is required for ectopic cell death, only the CC_EDVID_ of MLA10 has been shown to date to be sufficient for the induction of cell death signaling [16], [20], [24], [25]. Our truncation and mutagenesis analyses confirmed that the MLA10 CC domain is required and sufficient for triggering AVR-independent, but SGT1-/HSP90-dependent cell death, pointing to its role as a pro-cell death signaling domain (Figure 1, 2, 4, and S3). This activity is likely dependent on a unique surface charge segregation established upon CC homodimerization (Fig. 2 and [25]). Such a signaling function is in contrast to some studies that assign a specific role in pathogen recognition for the N-termini of CNL proteins [20], [39], [65], [66]. In the case of the Rx protein, conferring resistance against potato PVX, its NB domain was shown to mediate cell death signaling [39]. Thus, it appears that immune receptors of the CC_EDVID_-NB-LRR subclass engage distinct domains for triggering downstream cell death signaling.

What could be the role of the EDVID motif in MLA proteins? Our data show that this motif plays an important role in MLA10-triggered cell death signaling since it is needed for this activity of MLA10 FL and autoactive variants (both for the MHD and the F99E mutation; Figure 4B, 4C). While the EDVID motif in Rx appears to be involved in mediating intramolecular interactions between CC and the NB-ARC-LRR fragment [39], there is no direct evidence to support a similar function in MLA; nevertheless, molecular dynamics (MD) simulation of the MLA CC dimer shows that its EDVID motif could be involved in both intra- and intermolecular receptor interactions [25]. We could not resolve *in vivo* the role of the EDVID motif in MLA10 intramolecular interactions since we failed to detect an interaction between the CC and the NBARC-LRR of MLA10 by Co-IP, although we could reproduce the Rx intramolecular interactions in the *N. benthamiana* system (data not shown). An explanation might be that the interaction between MLA10 CC and NBARC-LRR domains is too weak/or transient to be detected. However, considering that the MLA10 CC does not only mediate MLA10 homodimerization but also associates with different molecules (for example the WRKY TFs, and other TFs according to our unpublished data) for downstream signaling, it is also possible that MLA10 undergoes different inter/intra-molecular interactions than those reported for other NB-LRR proteins.

Mutagenesis and biochemical studies have indicated that the NB-ARC domain acts as a molecular switch, whose status of nucleotide binding and hydrolysis is critical for regulating plant NB-LRR protein activity [12]–[14], [19]. We mutagenized the conserved P-loop and the MHD motifs in the MLA10 NB-ARC domain to examine their function in MLA10 cell death-inducing activity in both barley and *N. benthamiana* (Figure 3 and 4). Our data support the current NB-LRR protein activation model wherein the P-loop is involved in nucleotide binding and the MHD relays nucleotide-dependent conformational changes [10], [21]. Subsequently, we combined the P-loop mutation (K207R) with two MHD autoactivation mutations (H501A or D502V, respectively) to test the combined effect of double mutations on MLA10 cell death-inducing activity (Figure 4A). These MLA10 double mutants completely lost their cell death-inducing activity, consistent with the proposed model that MHD mutation-induced autoactivation mimics the nucleotide-dependent NB-LRR resistance protein activation that is normally triggered by pathogen-derived AVR effectors [19], [24]. It is possible that the MLA10 receptor autoactivation by MHD motif mutations needs bound NTP and that this NTP binding is compromised in conjunction with the mutated P-loop motif.

The MLA10 N-terminal fragments (CC, CC-NB, CC-NB-ARC) induce P-loop- or EDVID-independent cell death, whereas the MLA10 WT or autoactive FL mutant variants (both the MHD mutations or the F99E substitution) do require an intact P-loop and EDVID motif to induce cell death (Figure 4). These data suggest that the N-terminal fragments might mimic an MLA10 post-activation state that does no longer require the P-loop function. It also indicates that the C-terminal LRR region of MLA10 plays a negative regulatory role in MLA10 cell death-inducing activity. It has been observed for several other NB-LRR proteins that the LRR domain negatively regulates disease resistance activity [16], [20], [29], [33].

### The Role of Nuclear and Cytoplasmic MLA10 Pools in Cell Death Signaling

To dissect the relation between the MLA10 subcellular localization and cell death signaling activity, YFP fusions of MLA10 FL and the CC-NB fragment were examined for their subcellular localization. Not surprisingly, both fusions exhibited a similar nucleocytoplasmic distribution in *N. benthamiana* leaf cells (Figure 6, and S6). The utilization of NLS/NES and GR sequences allowed us to examine the individual functions of the MLA10 nuclear and cytoplasmic pools for cell death induction. Significantly, enforced retention of full-length MLA10 or CC-NB YFP fusions in the nucleus blocked its cell death-triggering activity (Figure 5C, 6A). This finding suggests that either this MLA10 activity is inhibited in the nucleus or the mere nuclear localization of MLA10 is insufficient for triggering cell death, or both. Conversely, enhanced cytoplasmic localization of MLA10 FL or the CC-NB fragment by fusing them to a NES signal clearly increased their cell death-inducing activities (Figure 5C, 6A). This enhanced receptor activity is likely attributable to enhanced nuclear export- over import-rates (Figure 5C, 6A), implicating the cytoplasm as compartment for MLA10 to initiate and/or amplify post-activation cell death signaling. Furthermore, cytoplasmic retention of YFP fusions of full-length MLA10 WT or two autoactive mutant variants did not block their cell death-inducing activity (Figure 6C), again suggesting that MLA10 triggers cell-death signaling in the cytoplasm. In this context, the failure of cytoplasmic CC-NB-YFP-GR to stimulate cell death in the absence of Dex possibly reflects its enforced physical association with the chaperone/folding machinery (i.e., direct binding between the GR hormone binding domain and HSP90; [67]). Along the same line, resumed cell death activation after Dex treatment demonstrates that CC-NB-YFP-GR is *per se* signaling competent, at least after its release from the cytoplasmic chaperone/folding machinery.

### Implications for MLA10 Activation and Signaling

We previously demonstrated that the MLA10-YFP-NES fusion protein was compromised for MLA10 disease resistance activity against the powdery mildew fungus due to depletion of the nuclear receptor pool [44], suggesting that initiation of disease resistance signaling demands either a threshold level of the receptor or prolonged residence in this compartment. Together with the finding that activated MLA10 interacts with *Hv*WRKY1/2 in the nucleus [44], we reasoned that enforced nuclear localization of the receptors should be capable of triggering disease resistance signaling. Indeed, upon transient expression in barley cells MLA10-YFP-NLS fusion was found to localize exclusively in the nucleus, and importantly both MLA10-YFP-NLS and MLA10-NLS fusions confer disease resistance against *Bgh* in barley (Figure 5A, 5B). In this context, it is worth noting that the activity of cell death induction of MLA10 was enhanced for MLA10-NES or CC-NB-YFP-NES fusions compared to the non-NES-tagged versions. This enhanced receptor activity is likely attributable to enhanced nuclear export over import rates (Figure 5C, 6A), implicating the cytoplasm as compartment for MLA10 to initiate and/or amplify post-activation cell death signaling. To integrate our findings in a model, we propose a bifurcation of MLA10 disease resistance and cell-death signaling in a compartment-dependent manner, thereby ‘uncoupling’ disease resistance signaling from cell death induction. In this model, the nuclear MLA10 pool triggers disease resistance signaling while the cytoplasmic MLA10 pool induces cell-death signaling. Two additional pieces of evidence support this model. First, the CC domain containing the L18E substitution has diminished cell death-inducing activity (Figure 2A) but FL MLA10 containing the same mutation retains full disease resistance activity against the cognate *Bgh* isolate [25]. This difference is not attributed to altered subcellular distribution since CC(L18E)-YFP and FL(L18E)-YFP localized to both nucleus and cytoplasm in *N. benthamiana* or barley leaf cells respectively, similar to that of other mutants, for example, the CC(F83E) or FL(K207R) P-loop mutants (Figure S2). Second, RAR1 is required for MLA10 disease resistance function but not for its cell death induction ([55]; Figure S3). Our model for MLA functional bifurcation in disease resistance and cell-death signaling appears distinct from Rx [50], [51]. Rx hyperaccumulation in the nucleus blocks not only cell-death but also resistance to PVX whereas MLA10 enforced nuclear localization suppresses only the cell-death signaling but not resistance to *Bgh*.

It has been shown that different types of plant disease resistance proteins can be activated at diverse locations, for example, at the membrane and in the cytoplasm or the nucleus or both [24], [35], [41], [48]–[50]. In this context, it is interesting that coexpression of AVR_A10_ with MLA10-NLS did not induce cell-death in *N. benthamiana* (Figure S5). It is possible that activated MLA10-NLS might be suppressed in the nucleus for triggering cell-death. It may also suggest that MLA10 activation by AVR_A10_ specifically occurs in the cytoplasm and thus MLA10-NLS could not be activated in the nucleus of *N. benthamiana* leaf cells. Unfortunately, we cannot test this possibility in *N. benthamiana* by coexpressing AVR_A10_ with MLA10-NES or MLA10-GR since these fusions themselves trigger cell-death.

Our model also implies an important role of nucleocytoplasmic distribution in both defense and cell-death signaling. One scenario would be that activated MLA distributes across the nuclear envelope so that a portion reaches its cytoplasmic destination to trigger cell death signaling whereas another portion associates with nuclear targets such as *Hv*WRKY1/2 to induce disease resistance responses. The notion that nucleocytoplasmic distribution is important for defense signaling is in line with other studies [42], [68]–[70], for example, the nucleocytoplasmic partitioning of potato Rx [48], [49], [68], [69], and the nucleocytoplasmic localization of several other plant immune receptors and immune regulators, e.g. tobacco N, Arabidopsis RRS1-R, RPS4 and snc1 [43], [45]–[47], [71], as well as Arabidopsis NPR1 and EDS1 [64], [72]. The recent studies linking EDS1-RPS4 interaction with immune receptor activation consolidate the notion that proper nucleocytoplasmic partitioning of immune regulators/receptors is important for coordinated defense responses and support the existence of cell compartment-specific functions for immune receptors [48], [49]. Future experimentation will be needed to resolve where MLA is activated inside host cells, directly or indirectly, and to identify more targets of activated MLA in the cytoplasm and the nucleus.

## Materials and Methods

### Plant Materials

Barley (*Hordeum vulgare* L.) cultivar Golden Promise plants were grown in a growth chamber under a 16 hrs/8 hrs, 20°C/18°C day/night cycle with 70% relative humidity. *Nicotiana benthamiana* plants were grown in greenhouse at 24±1°C with a 16 hrs light period.

### Plasmid Construction

All oligonucleotide primers used in this study (marked SW××) were purchased from Invitrogen Life Technologies (Beijing, China), and are listed in supplemental Table S1. The following gateway (GW) binary vectors derived from CTAPi [73] for providing C-terminal tagged fusions were used in this study: CTAPi-GW-3×HA, CTAPi-GW-YFP, CTAPi-GW-mYFP and CTAPi-GW-3×Myc. To create these vectors, either 3×HA, YFP, mYFP (monomeric YFP), or 3×Myc epitope tag followed by a stop codon was inserted into CTAPi at the unique *Spe*I site.

Construction of plasmid pUbi-MLA10-YFP for expression of MLA10-YFP fusion proteins under the control of the Ubiquitin promoter was described previously [44]. MLA10 MHD motif mutants (H501G/R/Q/V/A, D502V) and the P-loop mutant (K207R) were generated by overlap extension PCR [74], [75] using two sets of primer pairs indicated in supplemental Table S2 on template pUbi-MLA10-YFP. The consequent two PCR products were joined in a second amplification step using primer pairs SW09/SW10. A 1564-bp restriction fragment generated with *Age*I and *Bbs*I containing the desired mutation was used to replace the MLA10 wild-type sequence in pUbi-MLA10-YFP to obtain MLA10 variants FL(H501G), FL(H501R), FL(H501Q), FL(H501V), FL(H501A), FL(D502V) or FL(K207R) (FL for full-length). The MLA10 FL wild-type and its mutant variants were then transferred to the entry clone pDNOR201 by Gateway BP reactions (Invitrogen) and subsequently to the binary vector CTAPi-GW-3×HA to obtain C-terminally 3×HA-tagged MLA10 fusions.

MLA10 CC (wild-type/mutants), CC-NB, CC-NB-ARC, NB, NB-ARC, and NB-LRR fragments were amplified from pUbi-MLA10-YFP using primer pairs SW56/SW55, SW56/SW58, SW56/SW33, SW32/SW58, SW32/SW33, SW32/SW20, respectively. The single amino acid substitutions in MLA10 CC domain have been described before [25]. The resulting PCR products were cloned into pDNOR201 via a Gateway BP reaction and subsequently to CTAPi-GW-3×HA, generating C-terminally 3×HA-tagged MLA10 fragments and MLA10 CC mutant variants. MLA10 CC, CC variants or CC-NB fused with a C-terminal TAP, YFP, mYFP or 3×Myc epitope tag was constructed based on CTAPi or its derivative vectors as described above. *AVR*_A10_ coding sequences were amplified from the vector pPS1(*AVR*_A10_) [60] with primers SW106/SW107. The amplification products were cloned into pDNOR201 via a Gateway BP reaction and subsequently to CTAPi-GW-YFP to create CTAPi- AVR_A10_-YFP.

GR fusions were generated based on the vector pBI-GR [62]. YFP fragments were amplified by PCR using primer pairs SW80/SW75, and introduced into the *Xba*I/*Bam*HI sites of pBI-GR to create pBI-YFP-GR. YFP-GR and GR fragments were prepared by amplification from pBI-YFP-GR with primers SW80/SW81 and SW82/SW81, and inserted into the unique *Spe*I site of CTAPi, yielding a Gateway-compatible vector CTAPi-GW-YFP-GR and CTAPi-GW-GR. MLA10 FL, FL(F99E), FL(D502V) and MLA10 CC-NB fragments were then recombined into CTAPi-GW-YFP-GR and CTAPi-GW-GR via a Gateway LR reaction to obtain CTAPi-MLA10-YFP-GR, CTAPi-FL(F99E)-YFP-GR, CTAPi-FL(D502V)-YFP-GR, CTAPi-CC-NB-YFP-GR and CTAPi-MLA10-GR. For subcellular localization analysis, the SV40 T-Ag NLS (QPKKKRKVGG)/PK1 NES (NELALKLAGLDINK) or their mutant nls (QPKKTRKVGG)/nes (NELALKAAGADANK) were fused to the C-terminus of MLA10-YFP or CC-NB-YFP by PCR using overhanging primers. The cloning details are available upon request.

### Single-Cell Transient Gene Expression Assay

Single-cell transient gene expression assays using biolistic delivery of plasmid DNA into barley epidermal cells were described previously [28]. A reporter plasmid (pUbi-GFP) and plasmids expressing individual MLA10 or its mutant variants controlled by the Ubiquitin promoter were mixed before coating of gold particles (molar ratio of 1∶1; 2.5 µg of total DNA). Single leaf epidermal cells were transformed with a particle inflow gun of the model PDS-1000/He (Bio-Rad). In the experiments analyzing cell-death activity of MLA10 variants, all constructs were co-expressed with a GFP marker. The numbers of GFP expressing cells were scored at 36–42 hrs post bombardment using fluorescence microscope, which represents viable cells. The number of GFP cells, obtained after biolistic delivery of the control vector harboring functional MLA10-YFP and a GFP marker, were used as cell-death activity reference thus standardized to GFP Index equivalent to 1.000. GFP cell numbers from other constructs in the same experiments were standardized accordingly. To minimize the variations in the same experiments, same amounts of gold particles and DNA for coating were used and the barley leaves were also selected so that the leaf area to receive the particles was roughly same.

### Agrobacteria-Mediated Transient Expression

Agrobacteria-mediated transient expression (Agro-infiltration) using *Agrobacterium tumefaciens* strain GV3101 carrying the indicated constructs was performed as described by van Ooijen et al. [21]. Agrobacteria suspensions were infiltrated at OD_600_ = 0.5 (for HR inducing phenotype) or 1 (for protein isolation) into 4–5 weeks old *N. benthamiana* leaves.

### Trypan Blue Staining

*N. benthamiana* leaves were boiled for 5 min in a 1∶1 mixture of ethanol and staining solution (10 ml lactic acid, 10 ml glycerol, 10 g phenol and 10 mg trypan blue, dissolved in 10 ml distilled water) for staining. The leaves were then de-stained in 2.5 g ml^−1^ chloral hydrate in distilled water.

### Electrolyte Leakage Assays

Electrolyte leakage assays were performed as described previously [25] with following modifications. Briefly, four round leaf discs (11 mm in diameter) per measurement were cut from the infiltrated area at 22 hrs post Agro-infiltration, washed in ultrapure water for 30 min, and subsequently transferred to a 60 mm-Petri dish containing 10 ml of ultrapure water supplemented with 0.001% Silwet L-77. Electrolyte leakage was determined using a B-173 ion conductivity meter (Horiba) at the indicated time points. Means and error bars were calculated from three replicates per time point and construct. Analysis of variance (ANOVA) was performed using SPSS (Version 13.0), and the Tukey's honestly significant difference (HSD) test (*p*<0.05) was applied. Similar experiments were repeated at least twice.

### Subcellular Localization

Cell suspensions of *A. tumefaciens* strain GV3101 containing the indicated constructs were infiltrated into *N. benthamiana* leaves. Confocal images were taken at the indicated time points using a confocal laser-scanning microscope Zeiss LSM 710 (Carl-Zeiss). Induced release of YFP-GR protein fusions were induced by spraying the leaves with 30 µM DEX at 6 hrs post infiltration.

### Protein Analyses

For the protein accumulation analysis, leaf samples were collected just prior to cell death occurring. Total soluble proteins of three 11-mm-diameter leaf discs from the infiltrated area were extracted in 200 µl of 2× Laemmli buffer [76], boiled and centrifuged. Proteins from 20 µl of the obtained supernatant were separated via SDS-PAGE and HA-tagged protein fraction was detected by immunoblotting using rat anti-HA antibody (Roche, 11867423001) and anti-rat IgG conjugated with horseradish peroxidase (HRP) (Sigma, A5795) for HA-tagged proteins, mouse anti-c-Myc antibody (Sigma, M4439) and anti-mouse IgG conjugated with HRP (Sigma, A9044) for myc-tagged proteins, and mouse anti-GFP antibody (Roche, 11814460001) and anti-mouse IgG conjugated with HRP for YFP (mYFP)-tagged proteins, respectively. TAP-tagged proteins were detected using peroxidase anti-peroxidase soluble complex (Sigma, P1291). NLS/NES and their mutant nls/nes fused MLA10 proteins were detected by antibody raised against MLA27. Pierce ECL western blotting substrate was used for detection.

### Virus-Induced Gene Silencing

Tobacco rattle virus-based virus induced gene silencing (VIGS) was operated as described by Liu et al. [77] with some modifications. pTRV1 and pTRV2-derived constructs were transformed into *A. tumefaciens* strain GV3101. Agrobacteria were grown overnight in LB to nearly saturated, spun down, and resuspended in the same volume of infiltration buffer (10 mM MgCl_2_, 100 µM acetosyringone and 10 mM MES). Cell cultures containing pTRV1 were then mixed with those containing pTRV2-derived constructs in 1∶1 ratio before infiltration. Three or four fully expanded leaves of three-weeks-old *N. benthamiana* plants were infiltrated with the mixture. Three weeks after VIGS, these plants were infiltrated with cell suspensions of Agrobacteria containing the indicated constructs.

### RNA Isolation and RT-PCR Analysis

Total RNA of silenced and non-silenced *N. benthamiana* leaves were extracted from three independent biological replicates using TRizol solution (Invitrogen) and treated by RNase-free DNase I (Takara) to remove the potential DNA contamination. First-strand cDNA was synthesized using 2 µg of total RNA, Oligo(dT)18 and M-MLV Reverse Transcriptase (Promega). The cDNAs were then used as templates for Semi-quantitative RT-PCR using gene-specific primers outside the region targeted for silencing. For detection of *NbRAR1*, *NbSGT1* and *NbHSP90*, primers SW87/SW88, SW89/SW90 and SW91/SW92 were used, respectively.

### Gene Accession Numbers

Sequence data for genes used in this article can be found under GenBank accession numbers AY266445 (*Mla10*), AF494083 (*NbSGT1*), AY368904 (*NbHSP90-1*), AY368905 (*NbHSP90-2*), AY438026 (*NbRAR1*), and DQ679913 (*AVR_A10_*).

## Supporting Information

Figure S1 **Analysis of cell death inducing activity of epitope-tagged MLA10 fragments.** (**A**) Analysis of cell death inducing activity of MLA10 CC with various epitope tags. MLA10 CC alone or fused with different C-terminal tag (-3×HA, -3×Myc, -mYFP or -TAP) were expressed by agro-infiltration in *N. benthamiana* leaves, and cell-death triggered by each fusion was scored by trypan blue staining 48 hpi (left panel). Proteins were extracted from *N. benthamiana* leaves at 36 hpi and expression levels of individual fusions were assessed by immunoblotting with indicated antibodies (right). Asterisk indicates non-specific signals. TAP: tandem affinity purification. (**B**) Analysis of cell death inducing activity of MLA10 CC-NB with various epitope tags. MLA10 CC-NB alone or fused with different C-terminal tag (-3×HA, -TAP, -YFP or -mYFP) were expressed by Agro-infiltration in *N. benthamiana* leaves, and cell-death triggered by each protein was scored by trypan blue staining 48 hrs post Agro-infiltration (left). Proteins were extracted from *N. benthamiana* leaves at 36 hpi and expression levels of individual fusion proteins were assessed by immunoblotting with indicated antibodies (right).
(PDF)

Figure S2 **Subcellular localization of YFP fusions of MLA10 CC and FL mutant variants.** Indicated YFP fusions of MLA10 CC mutant variants were expressed respectively in *N. benthamiana* leaves by Agro-infiltration, and confocal images were taken at ∼24 hpi post infiltration (upper panel). Indicated YFP fusions of MLA10 FL variants were delivered into barley epidermal cells through particle bombardment, and confocal images were taken at ∼36 hrs post bombardment. Images represent *z*-stack 3D reconstruction (bottom panel). Scale bar equals to 50 µm.
(PDF)

Figure S3 **MLA10 cell death signaling activity is dependent on SGT1 and HSP90, but not RAR1.** (**A**) MLA10 cell death signaling in *Sgt1*-, *Rar1*- and *Hsp90*-silenced *N. benthamiana* plants. Lower *N. benthamiana* leaf was Agro-infiltrated with TRV vectors harboring fragments of *NbSgt1*, *NbRar1* or *NbHsp90* for respective silencing of these genes. Four weeks after TRV mediated silencing, indicated MLA10 fragments were expressed in the upper leaves of the silenced plants, and three days after agro-infiltration leaves were photographed (top row), and stained with Trypan blue for cell death phenotype (bottom row). (**B**) TRV vectors mediated silencing efficacy for *Sgt1*, *Rar1* and *Hsp90* in *N. benthamiana*. Agrose gel electrophoresis pictures show semi-quantitative RT-PCR products for *Hsp90*, *Sgt1* and *Rar1* using cDNA templates from non-silenced or silenced plants (upper panel), PCR cycle numbers were indicated above the gel picture, and *Actin* was included as an internal control (lower panel). The first strand cDNAs used as RT-PCR templates were synthesized from total RNA isolated from non-silenced (TRV:00) or silenced plants (TRV:Hsp90, TRV:Sgt1 and TRV:Rar1) using oligo (dT) primer and reverse transcriptase.
(PDF)

Figure S4 **Analysis of cell death Inducing activity of MLA10 fusions and mutant variants in barley.** The Plasmids of WT MLA10, a MHD motif mutant (D502V), a P-loop mutant (K207R) and two MLA10 fusions (MLA10-NLS and MLA10-NES) were co-expressed respectively with a GFP maker plasmid in barley epidermal cells using biolistic delivery. The histogram bars represent the number of GFP expressing cell of each co-expression experiment standardized to the MLA10-YFP as control (see M&M). GFP expressing cells were scored as vital cells at 36–42 hrs post bombardment. The error bars represented SE of three representing experiments.
(PDF)

Figure S5 **Analysis of cell death activity of AVR_A10_-YFP alone or coexpressing with MLA10-NLS.** AVRA10 and MLA10-NLS fusion were expressed alone or coexpressed in *N. benthamiana* leaf by Agro-infiltration. Confocal image of *N. benthamiana* cells expressing AVRA10-YFP fusion was taken at ∼36 hrs post infiltration (left panel). Trypan blue staining for cell death phenotype was done at 48 hpi (right panel). Scale bar equals to 50 µm.
(PDF)

Figure S6 **Comparisons for YFP signal intensity and cell death inducing activity between CC-NB-YFP and MLA10-YFP.** MLA10 CC-NB-YFP or MLA10-YFP were expressed respectively in *N. benthamiana* leaves by Agro-infiltration, and confocal images were taken at ∼20 hpi (upper panel) and cell-death triggered by each fusion protein was scored by trypan blue staining at ∼48 hpi (lower panel). Scale bar is 50 µm.
(PDF)

Figure S7 **Protein immunoblotting analysis of CC-NB fusion variants with different tags before and after Dex treatment.** Indicated MLA10 CC-NB fusions, CC-NB-YFP, CC-NB-YFP-GR, CC-NB-YFP-NES and CC-NB-YFP-NLS, were expressed respectively in *N. benthamiana* leaves by Agro-infiltration. Buffer with/or without Dex were sprayed onto *N. benthamiana* leaf surface at ∼36 hpi before samples were collected for crude protein extractions, and followed by immunoblotting with an anti-GFP antibody (upper panel). GR: steroid binding domain of the mammalian glucocorticoid receptor; Ponceau Red staining of Rubisco indicates equal loading (lower panel).
(PDF)

Figure S8 **Analyses of subcellular localization and cell death activity of MLA10-YFP-GR without Dex treatment.** MLA10-YFP-GR was expressed in *N. benthamiana* leaves by Agro-infiltration. Confocal images were taken at ∼24 hpi (left panel). Trypan blue staining was done at ∼48 hpi post Agro-infiltration to reveal cell-death phenotype (right panel). GR: Steroid binding domain of the mammalian glucocorticoid receptor. Scale bar equals to 50 µm.
(PDF)

Table S1 **Names and sequence of oligonucleotides used in this study.**
(DOC)

Table S2 **Primer sets used for point mutation in this study.**
(DOC)

Table S3 **Analysis of cell death inducing activity of MLA10 mutant variants in barley cells through transient gene expression assay.**
(DOC)
