# Supplementary figures and images for: Structure-Function Analysis of Barley NLR Immune Receptor MLA10 Reveals Its Cell Compartment Specific Activity in Cell Death and Disease Resistance
Source: PLoS Pathog. 2012 Jun 7;8(6):e1002752. doi: 10.1371/journal.ppat.1002752 (PMC3369952; doi:10.1371/journal.ppat.1002752)

Figure S1

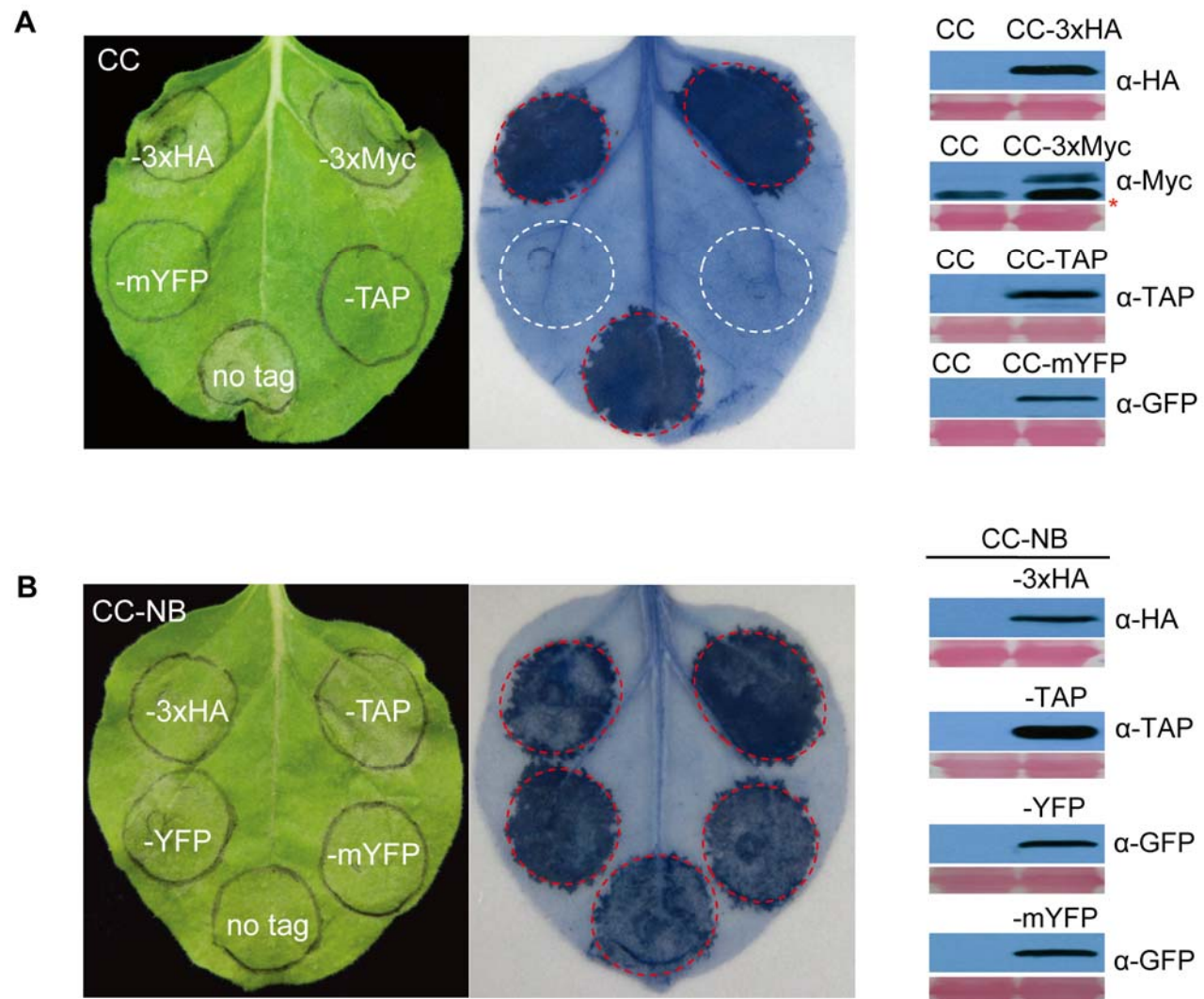

Supplement: Figure S1 — Analysis of cell death inducing activity of epitope-tagged MLA10 fragments. (A) Analysis of cell death inducing activity of MLA10 CC with various epitope tags. MLA10 CC alone or fused with different C-terminal tag (-3×HA, -3×Myc, -mYFP or -TAP) were expressed by agro-infiltration in N. benthamiana leaves, and cell-death triggered by each fusion was scored by trypan blue staining 48 hpi (left panel). Proteins were extracted from N. benthamiana leaves at 36 hpi and expression levels of individual fusions were assessed by immunoblotting with indicated antibodies (right). Asterisk indicates non-specific signals. TAP: tandem affinity purification. (B) Analysis of cell death inducing activity of MLA10 CC-NB with various epitope tags. MLA10 CC-NB alone or fused with different C-terminal tag (-3×HA, -TAP, -YFP or -mYFP) were expressed by Agro-infiltration in N. benthamiana leaves, and cell-death triggered by each protein was scored by trypan blue staining 48 hrs post Agro-infiltration (left). Proteins were extracted from N. benthamiana leaves at 36 hpi and expression levels of individual fusion proteins were assessed by immunoblotting with indicated antibodies (right). (PDF) [file ppat.1002752.s001.pdf]

Figure S2

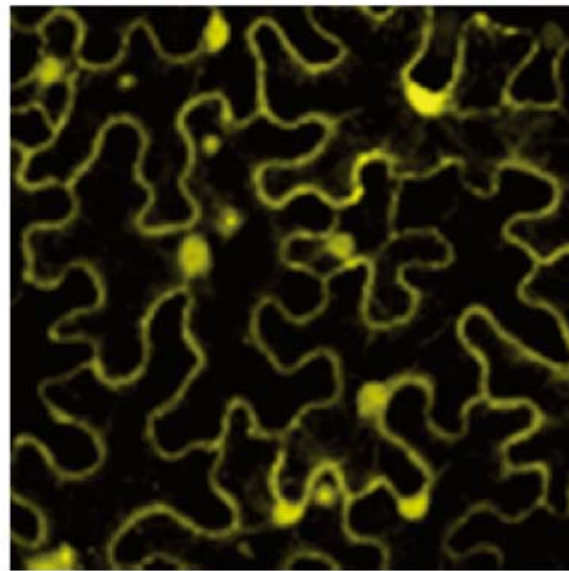

CC(L18E)-YFP

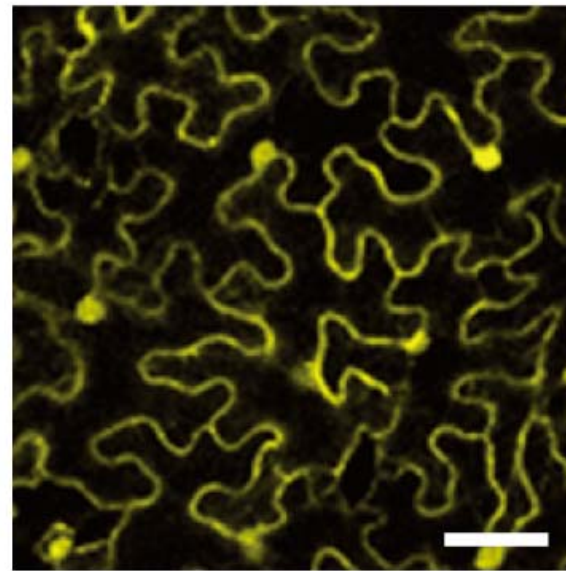

CC(F83E)-YFP

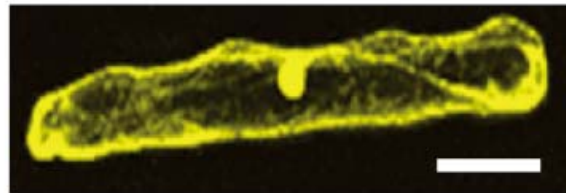

FL(L18E)-YFP

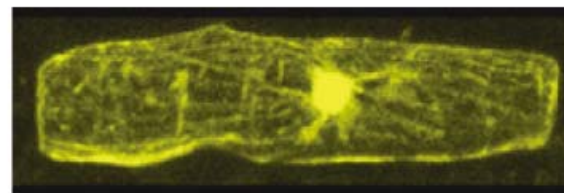

FL(K207R)-YFP

Supplement: Figure S2 — Subcellular localization of YFP fusions of MLA10 CC and FL mutant variants. Indicated YFP fusions of MLA10 CC mutant variants were expressed respectively in N. benthamiana leaves by Agro-infiltration, and confocal images were taken at ∼24 hpi post infiltration (upper panel). Indicated YFP fusions of MLA10 FL variants were delivered into barley epidermal cells through particle bombardment, and confocal images were taken at ∼36 hrs post bombardment. Images represent z-stack 3D reconstruction (bottom panel). Scale bar equals to 50 µm. (PDF) [file ppat.1002752.s002.pdf]

Figure S3

A

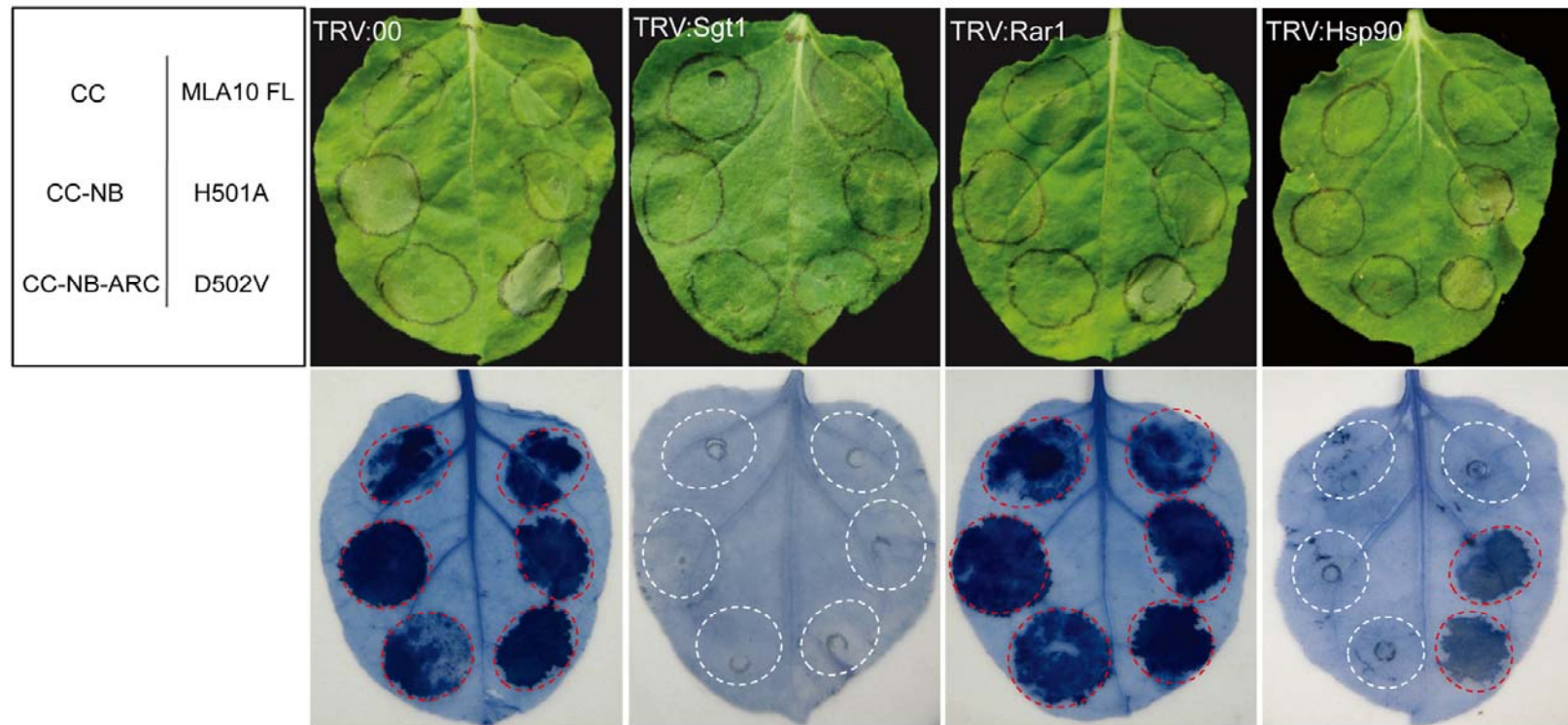

B

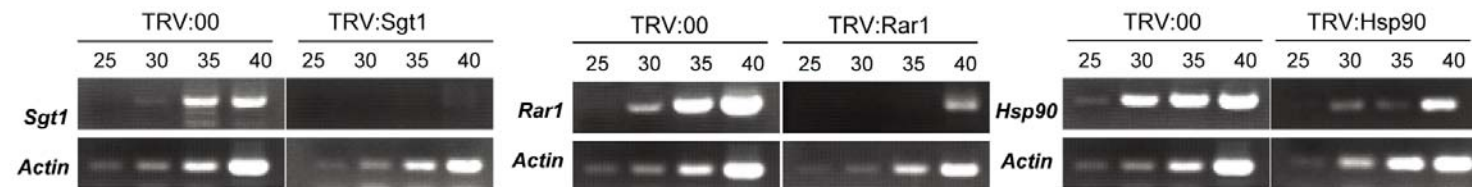

Supplement: Figure S3 — MLA10 cell death signaling activity is dependent on SGT1 and HSP90, but not RAR1. (A) MLA10 cell death signaling in Sgt1-, Rar1- and Hsp90-silenced N. benthamiana plants. Lower N. benthamiana leaf was Agro-infiltrated with TRV vectors harboring fragments of NbSgt1, NbRar1 or NbHsp90 for respective silencing of these genes. Four weeks after TRV mediated silencing, indicated MLA10 fragments were expressed in the upper leaves of the silenced plants, and three days after agro-infiltration leaves were photographed (top row), and stained with Trypan blue for cell death phenotype (bottom row). (B) TRV vectors mediated silencing efficacy for Sgt1, Rar1 and Hsp90 in N. benthamiana. Agrose gel electrophoresis pictures show semi-quantitative RT-PCR products for Hsp90, Sgt1 and Rar1 using cDNA templates from non-silenced or silenced plants (upper panel), PCR cycle numbers were indicated above the gel picture, and Actin was included as an internal control (lower panel). The first strand cDNAs used as RT-PCR templates were synthesized from total RNA isolated from non-silenced (TRV:00) or silenced plants (TRV:Hsp90, TRV:Sgt1 and TRV:Rar1) using oligo (dT) primer and reverse transcriptase. (PDF) [file ppat.1002752.s003.pdf]

Figure S4

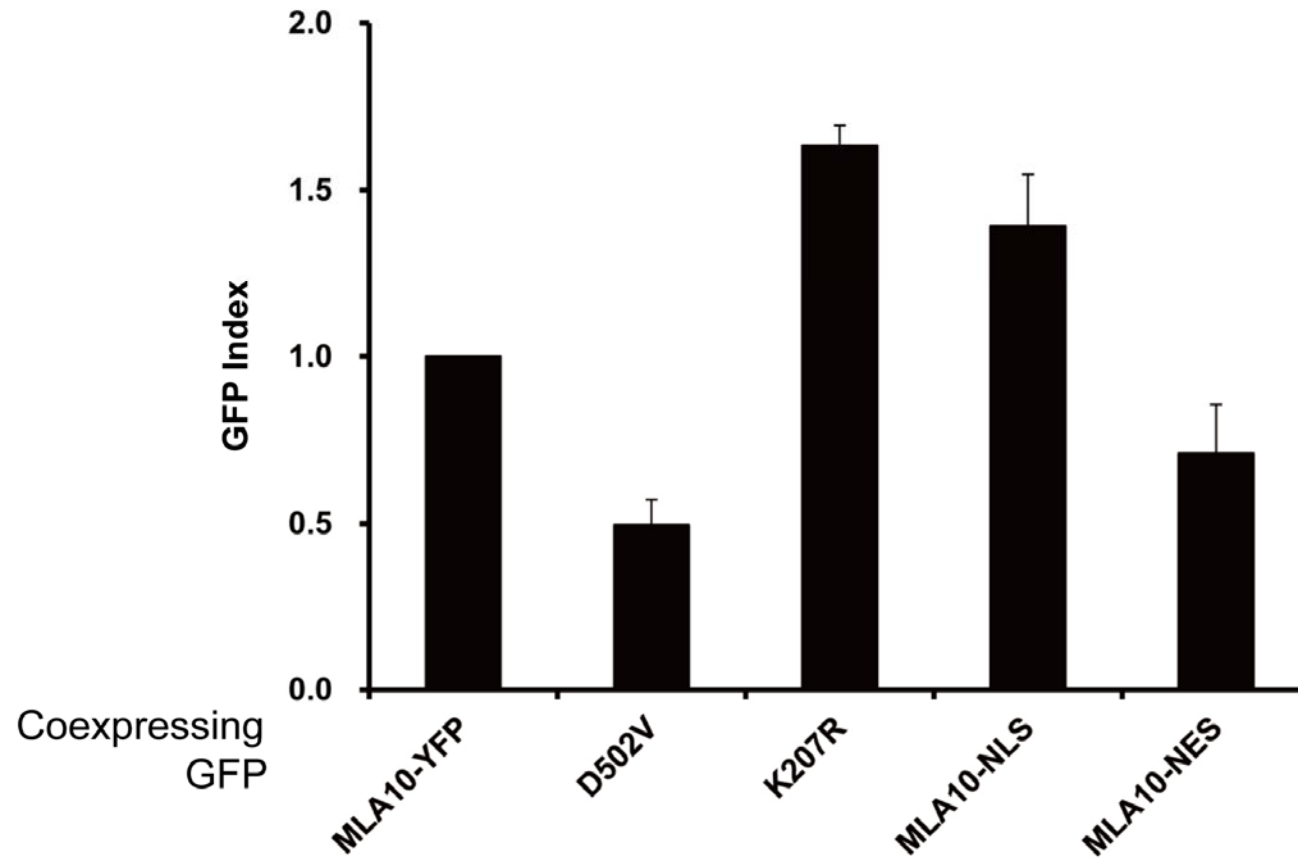

Supplement: Figure S4 — Analysis of cell death Inducing activity of MLA10 fusions and mutant variants in barley. The Plasmids of WT MLA10, a MHD motif mutant (D502V), a P-loop mutant (K207R) and two MLA10 fusions (MLA10-NLS and MLA10-NES) were co-expressed respectively with a GFP maker plasmid in barley epidermal cells using biolistic delivery. The histogram bars represent the number of GFP expressing cell of each co-expression experiment standardized to the MLA10-YFP as control (see M&M). GFP expressing cells were scored as vital cells at 36–42 hrs post bombardment. The error bars represented SE of three representing experiments. (PDF) [file ppat.1002752.s004.pdf]

Figure S5

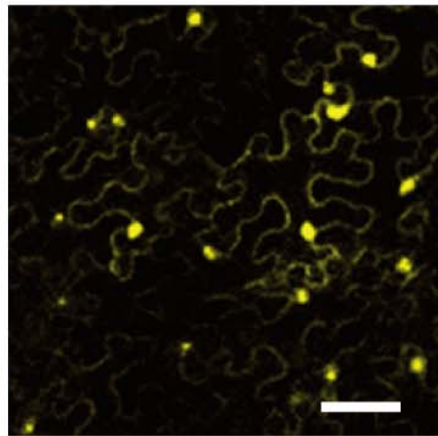

AVR<sub>A10</sub>-YFP

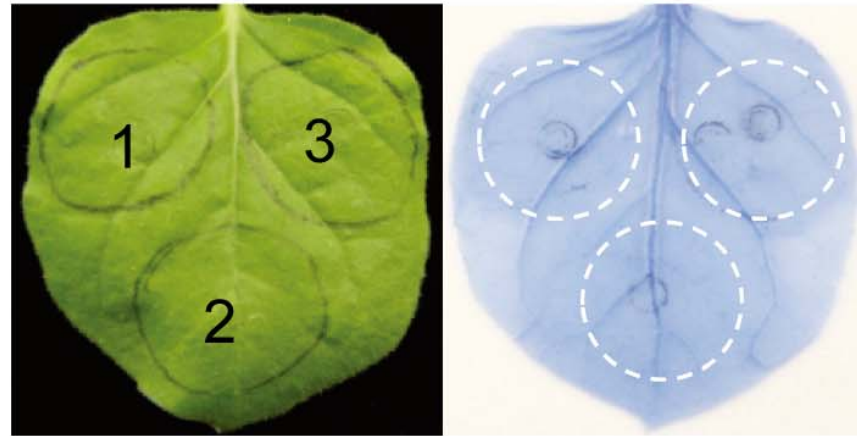

1. AVR<sub>A10</sub>-YFP
2. AVR<sub>A10</sub>-YFP + MLA10-NLS
3. MLA10-NLS

Supplement: Figure S5 — Analysis of cell death activity of AVRA10-YFP alone or coexpressing with MLA10-NLS. AVRA10 and MLA10-NLS fusion were expressed alone or coexpressed in N. benthamiana leaf by Agro-infiltration. Confocal image of N. benthamiana cells expressing AVRA10-YFP fusion was taken at ∼36 hrs post infiltration (left panel). Trypan blue staining for cell death phenotype was done at 48 hpi (right panel). Scale bar equals to 50 µm. (PDF) [file ppat.1002752.s005.pdf]

Figure S6

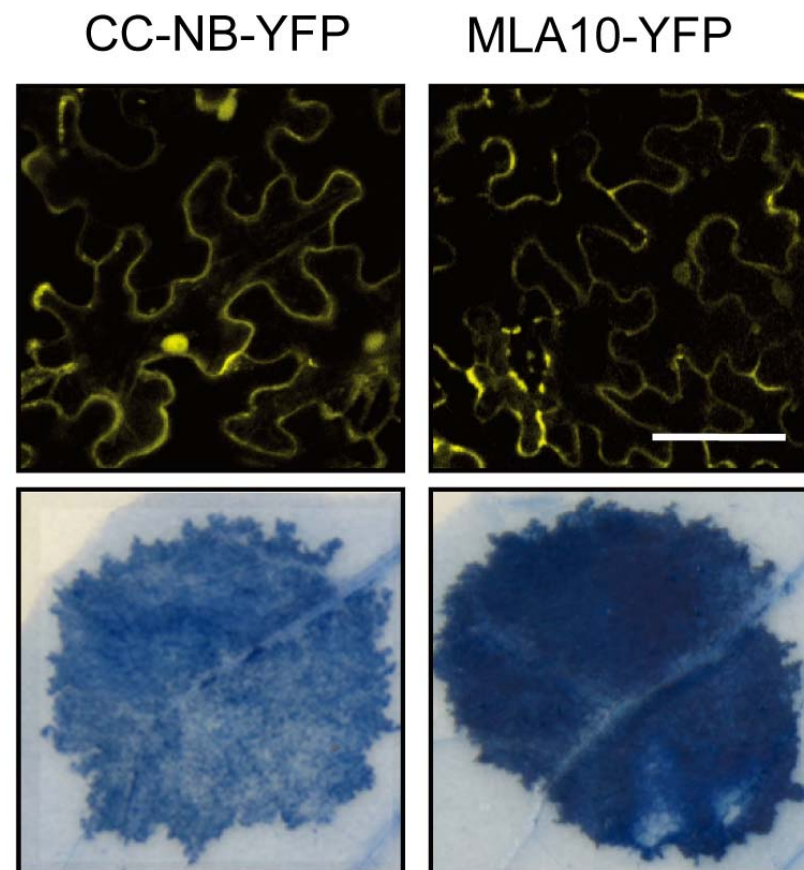

Supplement: Figure S6 — Comparisons for YFP signal intensity and cell death inducing activity between CC-NB-YFP and MLA10-YFP. MLA10 CC-NB-YFP or MLA10-YFP were expressed respectively in N. benthamiana leaves by Agro-infiltration, and confocal images were taken at ∼20 hpi (upper panel) and cell-death triggered by each fusion protein was scored by trypan blue staining at ∼48 hpi (lower panel). Scale bar is 50 µm. (PDF) [file ppat.1002752.s006.pdf]

Figure S7

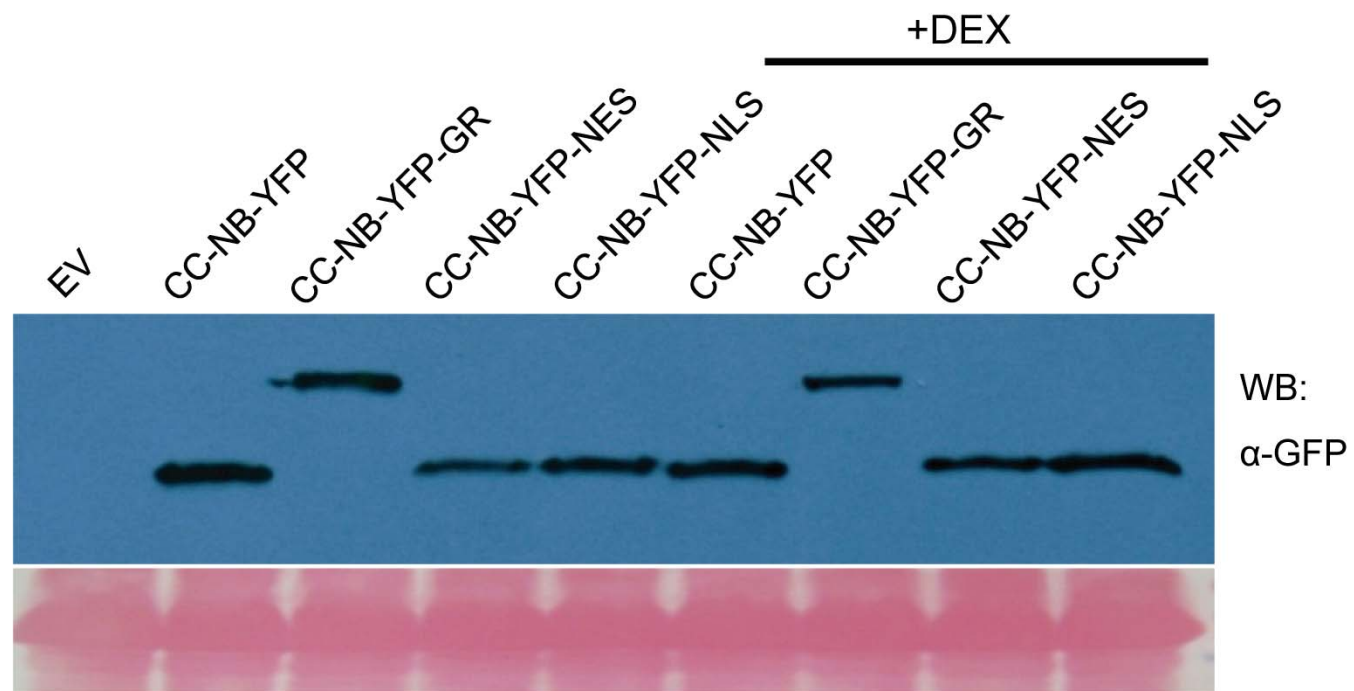

Supplement: Figure S7 — Protein immunoblotting analysis of CC-NB fusion variants with different tags before and after Dex treatment. Indicated MLA10 CC-NB fusions, CC-NB-YFP, CC-NB-YFP-GR, CC-NB-YFP-NES and CC-NB-YFP-NLS, were expressed respectively in N. benthamiana leaves by Agro-infiltration. Buffer with/or without Dex were sprayed onto N. benthamiana leaf surface at ∼36 hpi before samples were collected for crude protein extractions, and followed by immunoblotting with an anti-GFP antibody (upper panel). GR: steroid binding domain of the mammalian glucocorticoid receptor; Ponceau Red staining of Rubisco indicates equal loading (lower panel). (PDF) [file ppat.1002752.s007.pdf]

Figure S8

- DEX

---

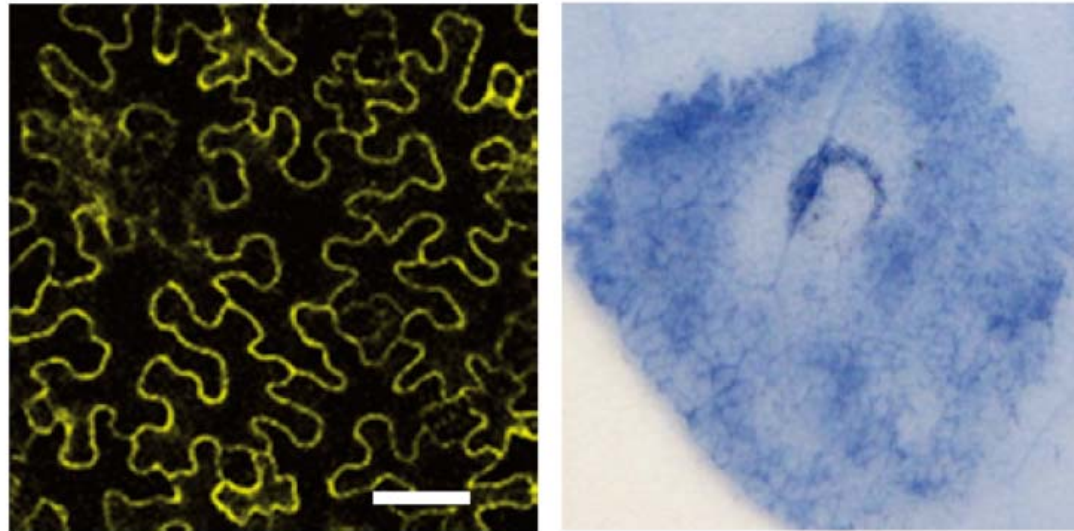

MLA10-YFP-GR

Supplement: Figure S8 — Analyses of subcellular localization and cell death activity of MLA10-YFP-GR without Dex treatment. MLA10-YFP-GR was expressed in N. benthamiana leaves by Agro-infiltration. Confocal images were taken at ∼24 hpi (left panel). Trypan blue staining was done at ∼48 hpi post Agro-infiltration to reveal cell-death phenotype (right panel). GR: Steroid binding domain of the mammalian glucocorticoid receptor. Scale bar equals to 50 µm. (PDF) [file ppat.1002752.s008.pdf]
