# Supplementary material for: Structure-Function Analysis of Barley NLR Immune Receptor MLA10 Reveals Its Cell Compartment Specific Activity in Cell Death and Disease Resistance
Source: PLoS Pathog. 2012 Jun 7;8(6):e1002752. doi: 10.1371/journal.ppat.1002752 (PMC3369952; doi:10.1371/journal.ppat.1002752)
Supplement: Table S1 — Names and sequence of oligonucleotides used in this study. (DOC) [file ppat.1002752.s009.doc]

Table S1. Names and Sequences of Oligonucleotides Used in this Study.

| Name | Sequence |
| --- | --- |
| SW01 | 5’-ccacgcagatattgtttga |
| SW05 | 5’-ggactgagctcctatcctccaacctttctcttcttcttaggctgcttgtacagctcgtccatgc |
| SW06 | 5’-ggactgagctcctatcctccaacctttctcgtcttcttaggctgcttgtacagctcgtccatgc |
| SW09 | 5’-ccctgttgtttggtgttactt |
| SW10 | 5’-caagatccaacacctccaaaaact |
| SW11 | 5’-gcttgccgtgtacgtgatatg |
| SW12 | 5’-ccagaaccatatcacgtacac |
| SW13 | 5’-gcttgccgtgtaggtgatatg |
| SW14 | 5’-ccagaaccatatcacctacac |
| SW15 | 5’-gcttgccgtgtagttgatatg |
| SW16 | 5’-ccagaaccatatcaactacac |
| SW17 | 5’-gcttgccgtgtacaagatatg |
| SW18 | 5’-ccagaaccatatcttgtacac |
| SW20 | 5’-ggggaccactttgtacaagaaagctgggtctaaatcgtcatcttgagcacc |
| SW32 | 5’-ggggacaagtttgtacaaaaaagcaggcttcatgtatgctgaagcgacagagctagttg |
| SW33 | 5’-ggggaccactttgtacaagaaagctgggtcaggcctggcttgatgatcttc |
| SW55 | 5’-ggggaccactttgtacaagaaagctgggtccaaagctcgaaggcaagggtc |
| SW56 | 5’-ggggacaagtttgtacaaaaaagcaggcttcatggatattgtcaccggtgc |
| SW58 | 5’-ggggaccactttgtacaagaaagctgggtcagaaagcggttccatttgataaac |
| SW75 | 5’- cgcggatcctccttgtacagctcgtccatgccg |
| SW80 | 5’-ctaggtctagaatggtgagcaagggcgaggagc |
| SW81 | 5’-ctaggactagttcatttttgatgaaacagaagc |
| SW82 | 5’-ctaggtctagagaagctcgaaaaacaaagaaaaaaatc |
| SW87 | 5’-gttgccagaggatcggttgca |
| SW88 | 5’-gcatggcgatatgctcatgaa |
| SW89 | 5’-agggaataccagccaagaat |
| SW90 | 5’-ccttctttgtgccaacttct |
| SW91 | 5’-tctccctgctgcttagtcac |
| SW92 | 5’-ttcctccatcttgctacctt |
| SW93 | 5’-ggagggttgggcaggaccactc |
| SW94 | 5’-gggcaggaccactcttgctagagc |
| SW95 | 5’-gcttgccgtgtacatgttatg |
| SW96 | 5’-ccagaaccataacatgtacac |
| SW97 | 5’-gcttgccgtgtagctgatatg |
| SW98 | 5’-ccagaaccatatcagctacac |
| SW106 | 5’-ggggacaagtttgtacaaaaaagcaggcttcatggactccagccattcaaaac |
| SW107 | 5’-ggggaccactttgtacaagaaagctgggtctgctgttgttgatgctgccggct |
