# Supplementary material for: Structure-Function Analysis of Barley NLR Immune Receptor MLA10 Reveals Its Cell Compartment Specific Activity in Cell Death and Disease Resistance
Source: PLoS Pathog. 2012 Jun 7;8(6):e1002752. doi: 10.1371/journal.ppat.1002752 (PMC3369952; doi:10.1371/journal.ppat.1002752)
Supplement: Table S2 — Primer sets used for point mutation in this study. (DOC) [file ppat.1002752.s010.doc]

**Table S2. Primer Sets Used for point mutation in this Study.**

| MLA10 variants | Primer sets | PCR product length (bp) |
| --- | --- | --- |
| FL(K207R) | SW09/SW94 | 750 |
|  | SW93/SW10 | 1300 |
| FL(H501G) | SW09/SW14 | 1620 |
|  | SW13/SW10 | 430 |
| FL(H501R) | SW09/SW12 | 1620 |
|  | SW11/SW10 | 430 |
| FL(H501Q) | SW09/SW18 | 1620 |
|  | SW17/SW10 | 430 |
| FL(H501V) | SW09/SW16 | 1620 |
|  | SW15/SW10 | 430 |
| FL(H501A) | SW09/SW98 | 1620 |
|  | SW97/SW10 | 430 |
| FL(D502V) | SW09/SW96 | 1620 |
|  | SW95/SW10 | 430 |
