# Supplementary material for: Structure-Function Analysis of Barley NLR Immune Receptor MLA10 Reveals Its Cell Compartment Specific Activity in Cell Death and Disease Resistance
Source: PLoS Pathog. 2012 Jun 7;8(6):e1002752. doi: 10.1371/journal.ppat.1002752 (PMC3369952; doi:10.1371/journal.ppat.1002752)
Supplement: Table S3 — Analysis of cell death inducing activity of MLA10 mutant variants in barley cells through transient gene expression assay. (DOC) [file ppat.1002752.s011.doc]

**Table S3. Analysis of cell death Inducing activity of MLA10 mutant variants in barley cells through transient gene expression assay**

| **Exp. 1** | | | | |  | **Exp. 2** | | | | |  |  | **Exp.3** | | | | |  |  |  |
| --- | --- | --- | --- | --- | --- | --- | --- | --- | --- | --- | --- | --- | --- | --- | --- | --- | --- | --- | --- | --- |
| **Vectors** |  | **GFP cell**  **No.** |  | **GFP**  **Index** |  | **Vectors** |  | **GFP cell**  **No.** |  | **GFP**  **Index** |  |  | **Vectors** |  | **GFP cell**  **No.** |  | **GFP**  **Index** |  | **Index**  **Avg.** | **SD** |
| **MLA10-YFP** |  | 90 |  | 1.000 |  | **MLA10-YFP** |  | 129 |  | 1.000 |  |  | **MLA10-YFP** |  | 134 |  | 1.000 |  | **1.000** | **0.000** |
| **H501R** |  | 33 |  | 0.367 |  | **H501R** |  | 57 |  | 0.442 |  |  | **H501R** |  | 73 |  | 0.545 |  | **0.451** | **0.089** |
| **H501G** |  | 39 |  | 0.433 |  | **H501G** |  | 61 |  | 0.473 |  |  | **H501G** |  | 83 |  | 0.619 |  | **0.509** | **0.098** |
| **H501Q** |  | 36 |  | 0.400 |  | **H501Q** |  | 59 |  | 0.457 |  |  | **H501Q** |  | 76 |  | 0.567 |  | **0.475** | **0.085** |
| **H501V** |  | 33 |  | 0.367 |  | **H501V** |  | 58 |  | 0.450 |  |  | **H501V** |  | 75 |  | 0.560 |  | **0.459** | **0.097** |
| **H501A** |  | 14 |  | 0.156 |  | **H501A** |  | 47 |  | 0.364 |  |  | **H501A** |  | 58 |  | 0.433 |  | **0.318** | **0.144** |
| **D502V** |  | 29 |  | 0.322 |  | **D502V** |  | 48 |  | 0.372 |  |  | **D502V** |  | 44 |  | 0.328 |  | **0.341** | **0.027** |
| **K207R** |  | 176 |  | 1.956 |  | **K207R** |  | 244 |  | 1.891 |  |  | **K207R** |  | 265 |  | 1.978 |  | **1.942** | **0.045** |

Notes: *Counting of GFP expressing cells starting from 36hpi. Avg.: average. SD: standard error
